# The Roles of Phosphorylation and SHAGGY-Like Protein Kinases in Geminivirus C4 Protein Induced Hyperplasia

**DOI:** 10.1371/journal.pone.0122356

**Published:** 2015-03-27

**Authors:** Katherine Mills-Lujan, David L. Andrews, Chau-wen Chou, C. Michael Deom

**Affiliations:** 1 Department of Plant Pathology, The University of Georgia, Athens, Georgia, United States of America; 2 Department of Chemistry, Proteomics and Mass Spectrometry Core Facility, The University of Georgia, Athens, Georgia, United States of America; University of Manitoba, CANADA

## Abstract

Even though plant cells are highly plastic, plants only develop hyperplasia under very specific abiotic and biotic stresses, such as when exposed to pathogens like *Beet curly top virus* (BCTV). The C4 protein of BCTV is sufficient to induce hyperplasia and alter *Arabidopsis* development. It was previously shown that C4 interacts with two *Arabidopsis* Shaggy-like protein kinases, AtSK21 and 23, which are negative regulators of brassinosteroid (BR) hormone signaling. Here we show that the C4 protein interacts with five additional AtSK family members. Bikinin, a competitive inhibitor of the seven AtSK family members that interact with C4, induced hyperplasia similar to that induced by the C4 protein. The Ser49 residue of C4 was found to be critical for C4 function, since: 1) mutagenesis of Ser49 to Ala abolished the C4-induced phenotype, abolished C4/AtSK interactions, and resulted in a mutant protein that failed to induce changes in the BR signaling pathway; 2) Ser49 is phosphorylated *in planta*; and 3) plant-encoded AtSKs must be catalytically active to interact with C4. A C4 N-myristoylation site mutant that does not localize to the plasma membrane and does not induce a phenotype, retained the ability to bind AtSKs. Taken together, these results suggest that plasma membrane associated C4 interacts with and co-opts multiple AtSKs to promote its own phosphorylation and activation to subsequently compromise cell cycle control.

## Introduction

Geminiviruses (family *Geminiviridae*) cause extensive agricultural losses in crops worldwide [1]. The family is divided into seven genera: *Becurtoviruses*, *Curtoviruses*, *Eragroviruses*, *Mastreviruses*, *Topocuviruses*, and *Turncurtoviruses* have monopartite genomes and *Begomoviruses* can have monopartite or bipartite genomes [2]. The viruses have small, circular, single-stranded DNA genomes (each ~2.5 to 3.0 kb) that do not encode a polymerase and must infect undifferentiated cells or reprogram differentiated cells to re-enter the S-phase of the cell cycle to access host-replication machinery for propagation [3,4]. Because of their unique features, geminiviruses represent a model system for studying plant cell cycle control, DNA replication, and gene expression [4,5].

Geminivirus-encoded Rep and RepA proteins bind to the host-encoded retinoblastoma-related protein (RBR) and interfere with the RBR-E2F transcriptional repression system, which controls entry into the S-phase of the cell cycle, to stimulate cell division or trigger endocycle [4,6,7]. Another geminivirus protein that induces cell proliferation is the C4 protein of some curtoviruses [8]. All geminiviruses, except mastreviruses, eragroviruses and becurtoviruses express a C4 protein, designated the AC4 protein in bipartite begomoviruses. The C4/AC4 proteins have diverse functions and are implicated in many different aspects of viral infection and pathogenicity; 1) transgenic expression of the curtovirus C4 gene induced hyperplasia in, and altered the development of, *Nicotiana benthamiana* [8] and *Arabidopsis* plants [9,10,11], 2) the C4 proteins of some curtoviruses and monopartite begomoviruses have been implicated in virus movement [11,12,13,14], and 3) the C4 proteins of some begomoviruses act as suppressors of gene silencing [15,16].

The role of the C4 protein has been enigmatic, but yeast two-hybrid studies showed that the *Beet curly top virus* (BCTV-B[US:Logan:76]) C4 protein and the *Tomato golden mosaic virus* (TGMV-[BR:Com:84]) AC4 protein interacted with two members of the *Arabidopsis* SHAGGY-like protein kinase family (AtSK21 and AtSK23), which are involved in the brassinosteroid (BR)-signaling pathway [17]. BRs are steroid hormones that promote plant growth and development. C4, and to a lesser extent AC4, were phosphorylated *in vitro* by AtSK21, suggesting a regulatory role for phosphorylation in C4/AC4 function [17]. Transgenic expression of the C4 protein in *Arabidopsis* plants alters the expression profiles of BR signaling pathway target genes, suggesting a direct interaction between C4 and the AtSKs involved in BR signaling [9]. Similarly, the C4 protein of *Tomato leaf curl virus* (ToLCV-To[AU]) was shown to interact with a novel SHAGGY-like protein kinase (SlSK) of tomato that is closely related to AtSK21 and AtSK23 [16]. More recently, silencing of a SHAGGY-like protein kinase (SK4-1/SSK) in *N*. *benthamiana*, a homologue of AtSK41, was shown to suppress *Tomato yellow leaf curl Sardinia virus* (TYLCSV-ES[ES:Alm2:92]) infection [18].

AtSKs are the *Arabidopsis* homologues of the evolutionarily conserved glycogen synthase kinase 3 family of serine/threonine kinases that play key regulatory roles in a wide range of signaling pathways [19,20]. AtSKs, which are encoded by a ten-member multigene family, are involved in regulating a diverse group of cellular processes including hormone signaling, development, and stress responses [21]. Based on the sequence comparison of their catalytic domains, AtSKs were divided into four subgroups: subgroup 1 (sg1AtSK11, -12, -13), subgroup 2 (sg2AtSK21, -22, -23), subgroup 3 (sg3AtSK31, -32) and subgroup 4 (sg4AtSK41, -42) [22].

Four of the ten AtSKs (sg2AtSKs and AtSK32) have been shown to act as negative regulators of BR signaling [23,24,25]. In the absence of BR, AtSKs hyperphosphorylate and inactivate the six members of the BES1/BZR1 family of transcription factors involved in BR-regulated gene expression [26,27]. However, in the presence of BR, the hormone binds to the BRI1 receptor kinase [28,29,30] and initiates a signaling cascade, which culminates in the dephosphorylation and inactivation of AtSKs involved in BR signaling [27,31]. The subsequent action of phosphatase 2A results in the accumulation of hypophosphorylated and active BES1/BZR1 family members [32]. Recent chemical genetic studies have identified a general AtSK antagonist, bikinin, which is an ATP competitor that specifically inhibits seven of the ten AtSKs (sg1AtSKs, sg2AtSKs, and AtSK32) [33].

Although recent evidence suggests that the BCTV C4-protein is activated by and/or interferes with one or more AtSK [9,17], little is known about the C4/AtSK interactions. In the present study, we identify amino acid residues in both C4 and AtSKs that are required for C4 function and C4/AtSK interactions. We show that C4 interacts strongly with seven AtSKs in yeast two-hybrid assays. *In planta* C4/AtSK interactions were shown to be dependent on a phosphoacceptor residue on C4 and an active kinase domain in the AtSKs. Bikinin-induced hyperplasia in *Arabidopsis* seedlings phenocopied C4-induced hyperplasia, suggesting that C4-induced hyperplasia may result from C4 interacting and interfering with the function of multiple AtSKs. Collectively, our results suggest that plasma membrane bound C4 is functionally activated via AtSK-mediated phosphorylation, which subsequently may inhibit the function of members of the AtSK family. In a manner recapitulated by bikinin, the interactions result in C4-induced hyperplasia and impaired development in *Arabidopsis*.

## Material and Methods

### Mutagenesis and mutant screen

The BCTV *C4* gene was amplified by polymerase chain reaction (PCR), as described [9] using primers PVXC45 and PVXC43 (S1 Table). The PCR product was cloned into pBSKS+ (Stratagene, La Jolla, CA) to give pBSKS-C4. Site-directed mutagenesis of *C4* was carried out using the QuikChange II Site-Directed Mutagenesis Kit (Stratagene, La Jolla, CA) with pBSKS-C4 as a template and the primers listed in the S2 Table. *C4* mutants (Table 1) were excised using *Cla*I and *Eco*RV and subcloned into the PVX-based expression vector pP2C2S [34] to give the pPVX-C4 mutant constructs. A non-translatable version of *C4* (*C4nt*) was also cloned into pP2C2S as above, using primers PVXC45 and C4NTE103, to give pPVX-C4nt. The integrity of *C4*, *C4nt* and the *C4*-mutants in all plasmids was confirmed by sequencing.

**Table 1:** C4 mutant phenotype screen on *N*. *benthamiana*.

**C4 mutant** ^1^	**Phenotype** ^2^	**C4 mutant**	**Phenotype**
**Acidic amino acid mutations**		**Serine/Threonine mutants**	
C4E41A	C4-like	C4S12A	C4-like
C4E56A	C4-like	C4S18A or T	C4-like ^4^
C4E69A	C4-like	C4S24A	C4-like
C4E73A	C4-like	C4S35A	C4-like
		C4S48A	C4-like
**Basic amino acid mutations**		C4S49A	PVX-like
C4K13A	C4-like ^3^	C4A49T	C4-like
C4R40A	C4-like	C4S52A	C4-like
C4R54A	C4-like	C4S66A	C4-like
C4R65A	C4-like	C4T38A	C4-like
C4R84A	C4-like	C4T47A	C4-like
C4R85A	C4-like	C4T51A	C4-like
		C4T55A	C4-like ^5^
**Double mutations**			
C4R40AE41A	C4-like	**N-myristoylation mutation**	
C4S52AR54A	C4-like	C4G2A	PVX-like

^1^ Mutant designation, point mutants: C4(Original amino acid)(residue position)(New amino acid).

^2^ Phenotype was scored as C4-like or PVX-like at 14 days post inoculation.

^3^ Milder C4-like, less contorted stem and less discoloration along leaf veins

^4^ Very mild phenotype, leaf surface is smooth, no stem curling or petiole rolling, only mild leaf curling on older leaves.

^5^ Symptoms C4-like, but onset was delayed 2 to 3 days.

RNA transcripts were obtained using the RiboMax Large Scale RNA Transcription System T7 (Promega, Madison, WI) and *Spe*I linearized pPVX-C4, pPVX-C4nt or pPVX-C4 mutant expression vectors as templates. Transcribed RNA was inoculated onto *N*. *benthamiana* leaves. Plants were kept in an environmentally controlled growth chamber (25°C, 16/8-h day/night cycles) and monitored for 14 days, phenotypes were noted, and tissue samples were assayed for the C4 protein by immunoblot analysis (see below).

### Generation of Transgenic *Arabidopsis* Plant Lines

*C4S49A* and *C4A49T* mutants were PCR-amplified using primers LOGAN45 and LOGANC43. *C4SII* and *C4A49TSII* were amplified using primers LOGAN45 and LOGANC43SII to fuse the StrepII peptide (SII) to the C-terminus of the C4 and C4A49T proteins, respectively. The PCR products were cloned into pER10, as previously described [9] to give pERC4S49A, pERC4A49T, pERC4SII and pERC4A49TSII. Genes cloned into pER10 are under regulatory control of a ß-estradiol-inducible promoter [35]. The plasmids were transformed into *Agrobacterium tumefaciens* strain ABI. *Arabidopsis thaliana* ecotype Sei-0 was used for transformation [36] and transformed plants were screened for single-copy insertion homozygotes, as previously described [9] to obtain plant lines IPC4S49A-2, IPC4A49T-2, IPC4SII-6 and IPC4A49TSII-1.

### Yeast two-hybrid assay

*C4* was PCR amplified using primers baitC45PR and BDC43PR. The PCR product was digested with *Nco*I and *Bam*HI and cloned into the multiple cloning site of the pGADT7 vector (Clontech, Mountain View, CA). The *C4*-mutants were cloned in a similar manner. The ten *AtSK* family members were PCR amplified from *Arabidopsis* ecotype Sei-0 cDNA, using the primers indicated in the S1 Table, cloned into either pGEM-T (Promega, Madison, WI) or pSCA-amp/kan (Stratagene, La Jolla, CA), verified by sequencing and then subcloned into the pGBKT7 vector (Clontech, Mountain View, CA) using appropriate restriction enzymes. The resulting pGBKT7-AtSK constructs were used for yeast transformations. Protein interactions in yeast were detected using the Matchmaker Two-Hybrid System 3 (Clontech, Mountain View, CA). Interactions were screened using the yeast strain AH109 co-transformed with activation domain (AD; pGADT7) and DNA binding domain (DBD; pGBKT7) constructs, using a 1:1 ratio of vector DNA. Co-transformants were grown in quadruple dropout medium (QDOM) lacking adenine, histidine, leucine, and tryptophan. Growth curves were generated using a Bioscreen C system (Growth Curves, Piscataway, NJ). Single colonies were grown overnight in standard Leu- Trp- dropout media. Cells were pelleted and washed 2X with QDOM, resuspended in QDOM and the OD_600_ was determined. Cell concentrations were adjusted to 0.15 OD_600_ in QDOM, 200 μL of each sample was loaded in wells of a 100-well sterile plate and cell suspensions were incubated for 3 days with shaking at 30°C. OD_600_ readings were automatically determined every 30 min. For each yeast two-hybrid time point, the average OD_600_ from three independent yeast transformants run in triplicate were used to generate growth curves using Bioscreener software (Growth Curves, Piscataway, NJ).

For quantification of β-galactosidase expression, yeast strain Y187 was co-transformed with C4-AD or C4 mutants-AD and the AtSKs-DBD constructs and selected on Leu- Trp- dropout medium. Quantitative analysis of ß-galactosidase expression was carried out on independent yeast transformants using the Galacto-Star System for yeast (Applied Biosystems Inc., Foster City, CA). Chemiluminescence was measured using a BioTek microplate reader (BioTek, VT). For each AD/DBD construct combination, three independent yeast transformants were assayed in triplicate. ß-galactosidase activity was calculated relative to the C4/AtSK23 interaction.

### Bimolecular fluorescence complementation

*C4*, *C4S49A*, and *C4A49T* were fused to portions of the enhanced yellow fluorescent protein (EYFP) gene. The genes were amplified by PCR using primers C4-EYFP 5F and C4-EYFP 3R, cloned into pBSKS+, and subsequently subcloned into the pSAT1-cEYFP-N1 vector [37] using *Nco*I and *Bam*HI restriction sites to give pC4-cEYFP, pC4S49A-cEYFP, and pC4A49T-cEYFP. *C4G2A* was amplified by PCR using the C4G2ANco and C4-EYFP 3R primer pair (S1 Table) and cloned into pSAT1-cEYFP-N1 to give pC4G2A-cEYFP. The resulting plasmids placed *C4* and the *C4*-mutants (lacking the endogenous stop codon) in-frame upstream of the C-terminal portion of EYFP (cEYFP). *C4* was similarly cloned into pSAT1-nEYFP-N1, which fuses *C4* in-frame upstream of the N-terminal portion of EYFP (nEYFP) to give pC4-nEYFP. The *AtSK*s in pGEM-T (see above) were subcloned into the multiple cloning site of either pSAT4-nEYFP-N1, for *AtSK21*, or pSAT4A-nEYFP-N1 for all other *AtSK*s [37]. In all cases, *AtSK*s (lacking the endogenous stop codon) were in-frame upstream of the N-terminal portion of EYFP. The fusion protein cassettes were subcloned into the pPZP-RCS2-bar vector [37] using *Asc*I for pSAT1-cEYFP-N1 clones and *I-Sce*I for pSAT4/4A-nEYFP-N1 clones. The integrity of the genes in all plasmids was confirmed by sequencing. All pPZP-RCS2 clones were subsequently transformed into *A*. *tumefaciens* strain LBA4404.

Fusion proteins were transiently expressed in transgenic *N*. *benthamiana* line CFP-H2B [38] following *A*. *tumefaciens* infiltration. Individual *A*. *tumefaciens* colonies were grown to stationary phase in LB broth with antibiotics. Cells were sub-cultured to an OD_600_ of between 0.8 and 1.0 in L-MESA medium (LB broth, 0.01 μM MES pH 5.7, 20 μM acetosyringone). Cells were harvested by centrifugation for 10 minutes at 3,500 x g, resuspended in agroinduction medium (0.01 μM MgCl_2_, 0.02 μM MES pH 5.7, 100 μM acetosyringone in water) to an OD_600_ between 1.0 and 1.1, and incubated at room temperature for 3-h. Cultures were combined at 1:1 ratios and infiltrated into the abaxial surface of young fully expanded *N*. *benthamiana* leaves using a 3 ml syringe without a needle. Infiltrated plants were incubated for 48-h at 25°C, 16/8-h day/night cycles. Water-mounted leaf tissue sections were examined with a Zeiss LSM 510 Meta confocal microscope equipped with a Zeiss Axio Imager M1 upright microscope and lasers spanning the spectral range from 405–514 nm (AHRC, University of Georgia). Images were acquired at a resolution of 2048 x 2048 pixels, with a scan rate of 1.6 ms pixel^-1^. Each plasmid combination was assayed three independent times.

*C4-nEYFP* or *C4-cEYFP* were PCR amplified (as above) using primer PVXC45 in combination with cEYFPEcoRev or nEYFPEcoRev. The resulting PCR products were digested with *Cla*I and *EcoR*V restriction sites, cloned into pP2C2S, linearized with *Spe*I, and transcribed RNA was inoculated into *N*. *benthamiana* plants as indicated above (see Mutagenesis and mutant screen section above).

### Protein and RNA analysis

Seedlings were germinated in liquid culture and grown for 7 days prior to induction with ß-estradiol, 1 μM brassinolide (BL) or both and harvested at 24 hours post-induction (hpi) [9]. C4 proteins were extracted, separated by sodium dodecyl sulfate polyacrylamide gel electrophoresis (SDS-PAGE), and blotted onto nitrocellulose as previously described [9]. BES1 was separated in 10% Criterion Tris-glycine gels (Bio-Rad, Hercules, CA) and blotted onto nitrocellulose. The C4 protein was detected using affinity purified C4 rabbit antiserum [9] and the BES1 protein was detected using BES1 antiserum [39] as primary antibodies. AtSK-nEYFPs were separated on 7.5% Criterion Tris-glycine gels. AtSK-nEYFPs and C4-cEYFP were detected using GFP antiserum (ab290) (Abcam, Cambridge, MA). An anti-rabbit polyclonal antiserum conjugated to alkaline phosphatase was used as a secondary antibody for visualization of C4 proteins and AtSK-nEYFPs (ProtoBlot II AP System, Promega, Madison, WI). Horseradish peroxidase-linked secondary antibodies were used for detecting BES1 with a SuperSignal Pico West kit (Thermo Fisher Scientific, Rockford, IL) followed by exposure to Hyperfilm^TM^ ECL X-ray film (Amersham Biosciences, Piscataway, NJ).

Quantitative real-time PCR (qRT-PCR) analysis was performed as previously described [9] in optical 96-well plates with an ABI 7500 real-time PCR system (Foster City, CA, USA). Relative expression levels were normalized to the ACT2 gene.

### Affinity purification

IPC4SII-6 or IPC4A49TSII-1 transgenic plants were grown in liquid culture in the presence or absence of ß-estradiol as described above. Protein was extracted from 1.5 g of transgenic plant tissue [40], but with a modified extraction buffer supplemented with PhosSTOP phosphatase inhibitor cocktail (Roche, Indianapolis, IN). C4SII and C4A49TSII were purified on gravity flow Strep-Tactin Sepharose columns following the manufacturer’s instructions (IBA GmbH, Goettingen, Germany) except that the washing, elution and regeneration buffers were supplemented with 0.5% Triton X-100. Eluted proteins were precipitated by addition of trichloroacetic acid to a final concentration of 10% (vol/vol) followed by centrifugation. The protein pellet was washed 2x with ice-cold acetone and dissolved in Tricine sample buffer (200 mM Tris-HCl, pH 6.8, 2% SDS, 40% glycerol, 2% ß-mercaptoethanol, 0.04% Coomassie Brilliant Blue G-250) at 95°C for 5 min. Proteins were resolved on Criterion Tris-Tricine/Peptide gels (Bio-Rad, Hercules, CA), stained with Bio-Safe Coomassie and destained according to the manufacturer’s direction (Bio-Rad, Hercules, CA).

### Mass spectrometry

Gel bands containing C4SII or C4A49TSII were sliced into small pieces and destained with 20 mM ammonium bicarbonate. Proteins were reduced with 100 μL of 10 mM dithiothreitol, alkylated with 100 μL of 100 mM iodoacetamide and digested overnight with 0.1 μg of trypsin in 20 mM ammonium bicarbonate at 37°C. The peptides were extracted with 0.1% TFA in 50% acetonitrile and dried in a SpeedVac. Peptides were resuspended in 10 μL of 0.1% formic acid/5% acetonitrile prior to LC-MS/MS analyses. A Thermo-Fisher LTQ Orbitrap Elite Mass Spectrometer coupled with a Proxeon Easy NanoLC system (Waltham, MA) was used for analysis (Proteomics and Mass Spectrometry Facility, University of Georgia).

Proteome Discoverer 1.3 (Thermo-Fisher) software was used with Mascot and SEQUEST database search engines for peptide and phosphorylation site identification. A custom database incorporates C4S49T, curtovirus proteins, and *A*. *thaliana* from the Uniprot database release 2013_02. Probabilities of phosphorylation sites were evaluated first by the phosphoRS tool of Proteome Discoverer followed by manual inspection to validate all possible sites.

All MS and MS/MS scans were performed in the Orbitrap at a resolution of 120,000 and 3,000 with a 5–7 ppm mass accuracy. Qual Browser of Xcalibur software from Thermo-Fisher was used for data analyses. The sequences of the selected ions were validated by their MS/MS spectra. Three independent C4SII and C4A49TSII purified protein preparations were analyzed by MS and each preparation was analyzed 2–4 times.

## Results

### C4 interacts with multiple AtSKs

**Fig 1:**
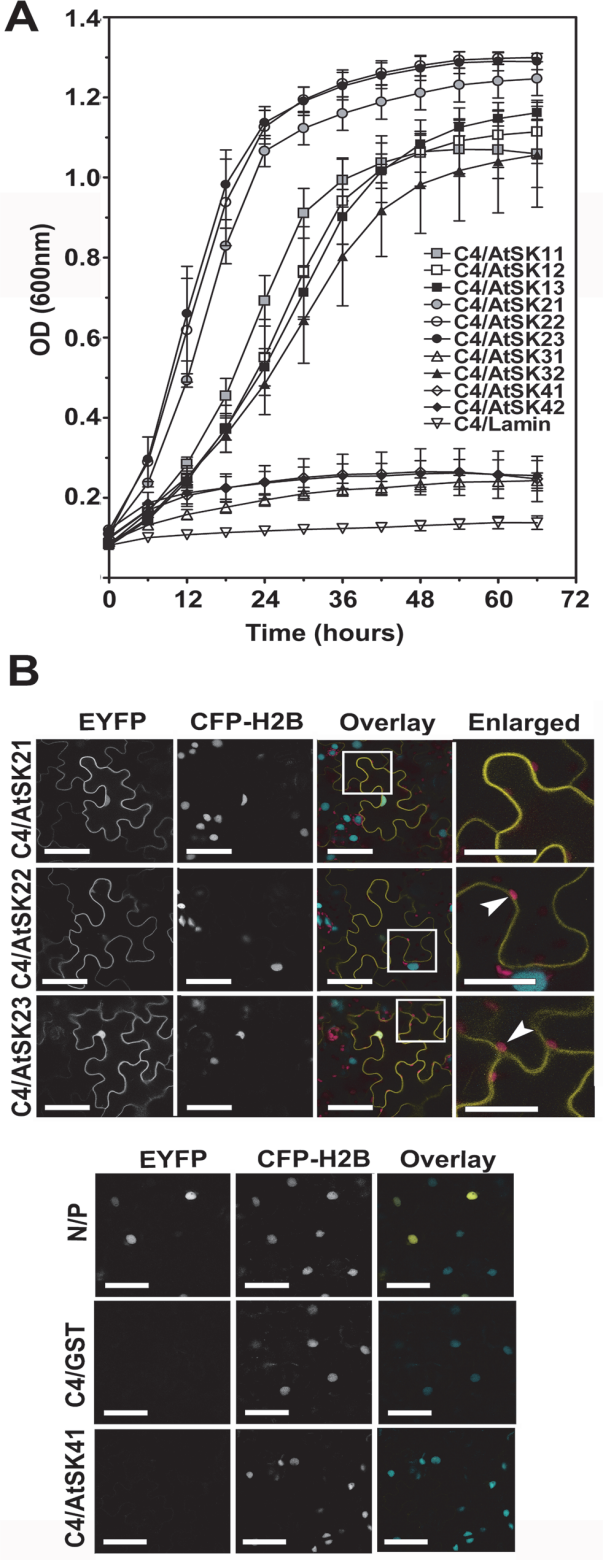
C4 interacts with multiple members of the *Arabidopsis* SHAGGY-like protein kinase family. A. Yeast two-hybrid growth curve analysis of BCTV C4 interactions with 10 AtSK family members. C4 was fused to the GAL4 activation domain and the AtSK family members and Lamin C (negative control) were fused to the GAL4 DNA binding domain. Cells were grown in quadruple dropout media at 30°C and their optical densities at A_600_ were measured. Each time point for each curve was obtained from three independent biological replicates run in triplicate. B. Confocal micrographs showing the interactions between C4-cEYFP and sg2AtSK-nEYFPs or AtSK41-nEYFP in BiFC assays following co-agroinfiltration into transgenic *N*. *benthamiana* CFP-H2B marker plants. From left to right, the first column shows the interaction assay EYFP signal (EYFP), the second the nuclear CFP-H2B reference signal (CFP-H2B), and the third the merger of both fluorescent signals with autofluorescence from chloroplasts (Overlay). Regions of co-localization appear yellow in the overlay. The known nYFP-N and cYFP-P interaction was used as a positive control [38] and GST-nEYFP [17] and C4-cEYFP were used as negative controls. Areas within the white boxes in the Overlay images are magnified in the Enlarged images. Arrowhead, chloroplasts. Scale bar = 50 μm.

C4 was previously shown to interact with two sg2AtSKs, AtSK21 and AtSK23 [17]. To investigate whether C4 interacts with additional members of the AtSK family, we performed yeast two-hybrid assays with the ten AtSK family members. C4/AtSK interactions were assayed using growth curves performed under high stringency conditions (Fig 1A). The lack of interaction between C4 and Lamin C was used as a negative control. C4 interacted not only with AtSK21 and AtSK23 as previously reported [17], but C4 also interacted with AtSK22, sg1AtSKs and AtSK32. In contrast, no significant interactions were detected between C4/AtSK31 or C4/sg4AtSKs (Fig 1A).

To determine if the interactions seen in the yeast two-hybrid assay could be detected *in planta*, we performed bimolecular fluorescence complementation (BiFC) assays [37,41,42]. C4 was C-terminally tagged, to avoid interference with the functional N-myristoylation motif [17], with either the N-terminal portion (C4-nEYFP) or the C-terminal portion (C4-cEYFP) of EYFP, and assayed for functionality in *N*. *benthamiana*. The phenotype induced by the C4-cEYFP protein was similar to the wild type C4 protein phenotype and was subsequently used in BiFC assays, while the C4-nEYFP protein induced a less severe phenotype (S1A Fig). Previously, studies using a C-terminally tagged AtSK21 showed little, if any, affect on function [43]. Therefore, sg2AtSKs, which interacted strongly with C4 in yeast two-hybrid assays, and AtSK41, which failed to interact with C4 in yeast two-hybrid assays, were used to examine *in planta* interactions with C4. Individual AtSK-nEYP fusion proteins were co-agroinfiltrated with C4-cEYFP into transgenic *N*. *benthamiana* plant line CFP-H2B (hereafter referred to as CFP-H2B), which constitutively expresses a cyan fluorescent protein (CFP) fused to histone 2B that localizes to the nucleus and serves as a spatial reference [38]. The nuclear localized interaction between the N and P proteins of *Sonchus yellow net virus* (SYNV) fused to nYFP and cYFP, respectively, served as a positive control [38; Fig 1B; Overlay image]. The nEYFP-GST fusion protein co-infiltrated with C4-cEYFP was used as a negative control [17; Fig 1B].

C4-cEYFP interacted with all sg2AtSK-nEYFPs when co-infiltrated into CFP-H2B plants (Fig 1B). The C4/sg2AtSK interactions occurred primarily at the plasma membrane, with some EYFP signal also detectable in the nucleus, as evident in the overlay images of the EYFP and CFP signals (see C4/AtSK21 and C4/AtSK23 Overlay and Enlarged images). As with the yeast two-hybrid assay, no interaction was detected between C4 and AtSK41 (Fig 1B) in BiFC assays, although co-expression of C4-cEYFP and AtSK41-nEYFP in inoculated CFP-H2B plants was confirmed by immunoblot analysis (S2 Fig). Localization of C4-EYFP (intact EYFP fused to the C-terminus of C4) was identical to that detected for the C4-cEYFP/sg2AtSK-nEYFP interactions (compare S1B Fig with Fig 1B), whereas AtSK21, -22, and -41-mCherry fusions localized, in a similar pattern to that previously described for AtSK21 (23), to the nucleus, cytoplasm and the plasma membrane (S1C Fig).

### C4-induced hyperplasia in sg2AtSK triple mutant and hypophosphorylation of BES1

**Fig 2:**
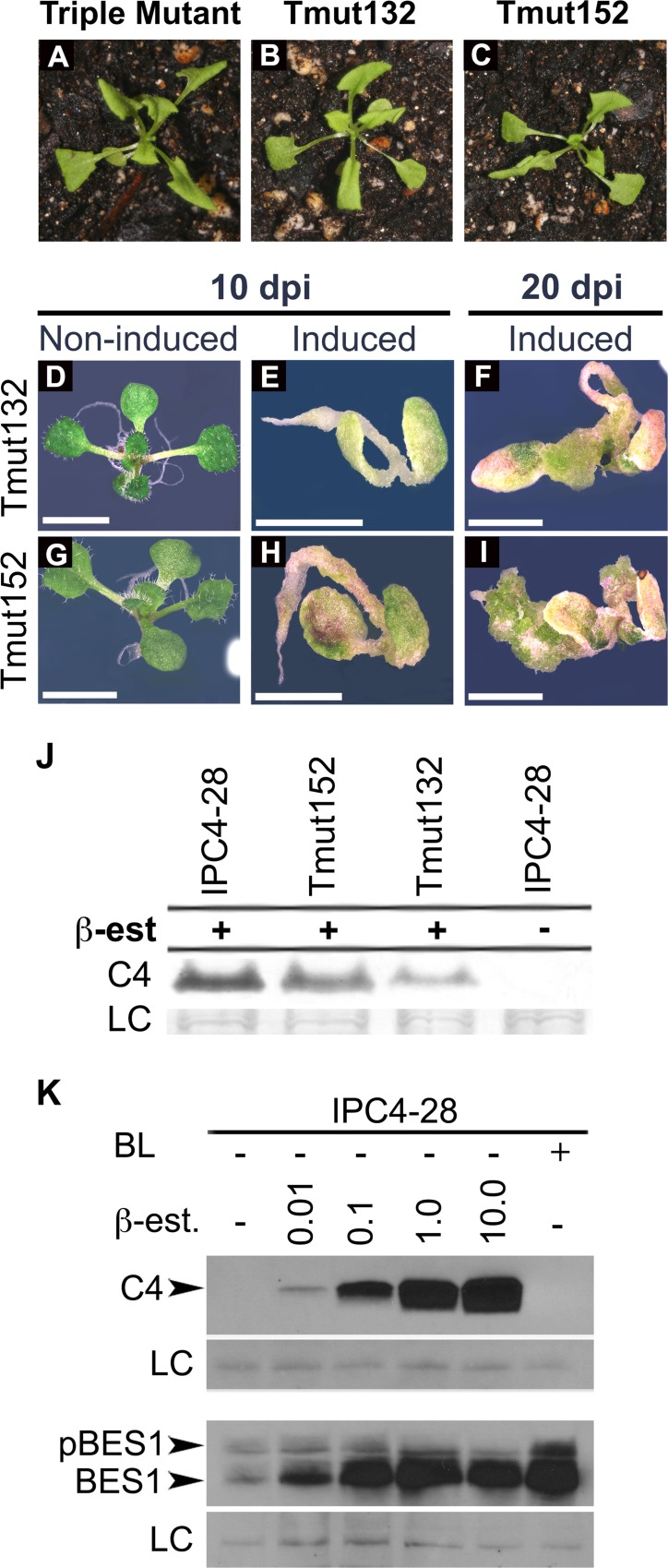
C4 expression induces hyperplasia in triple mutant seedlings and inhibits BES1 phosphorylation in wild-type seedlings. A-I. The sg2AtSK triple mutant and two independently derived transgenic triple mutant *Arabidopsis* lines, Tmut132 and Tmut152, expressing C4 under the control of a ß-estradiol inducible promoter were germinated and grown in soil (A-C), on solid media (D,G), or solid induction media at 10 days post-induction (E,H) and 20 days post-induction (F,I). J. IPC4-28, Tmut132 and Tmut152 seedlings were germinated and grown for 7 days in liquid media prior to induction with 5 μM of ß-estradiol, collected 24 hpi, and extracted proteins were assayed by immunoblot analysis. K. IPC4-28 seedlings were germinated and grown for 7 days in liquid media prior to induction with increasing concentrations of ß-estradiol (0.0, 0.01, 0.1, 1.0, 10.0 μM), BL only or non-induced, collected 24 hpi, and extracted proteins were assayed by immunoblot analysis. Levels of C4 shown in upper panel with corresponding shift in BES1 phosphorylation status shown in lower panel. Non-specific protein used as a loading control (LC) is shown below each immunoblot. BL, brassinolide; ß-est., ß-estradiol; phosphorylated BES1, pBES1; hypophosphorylated BES1, BES1. Scale bars = 4 mm (D-I).

**Fig 3:**
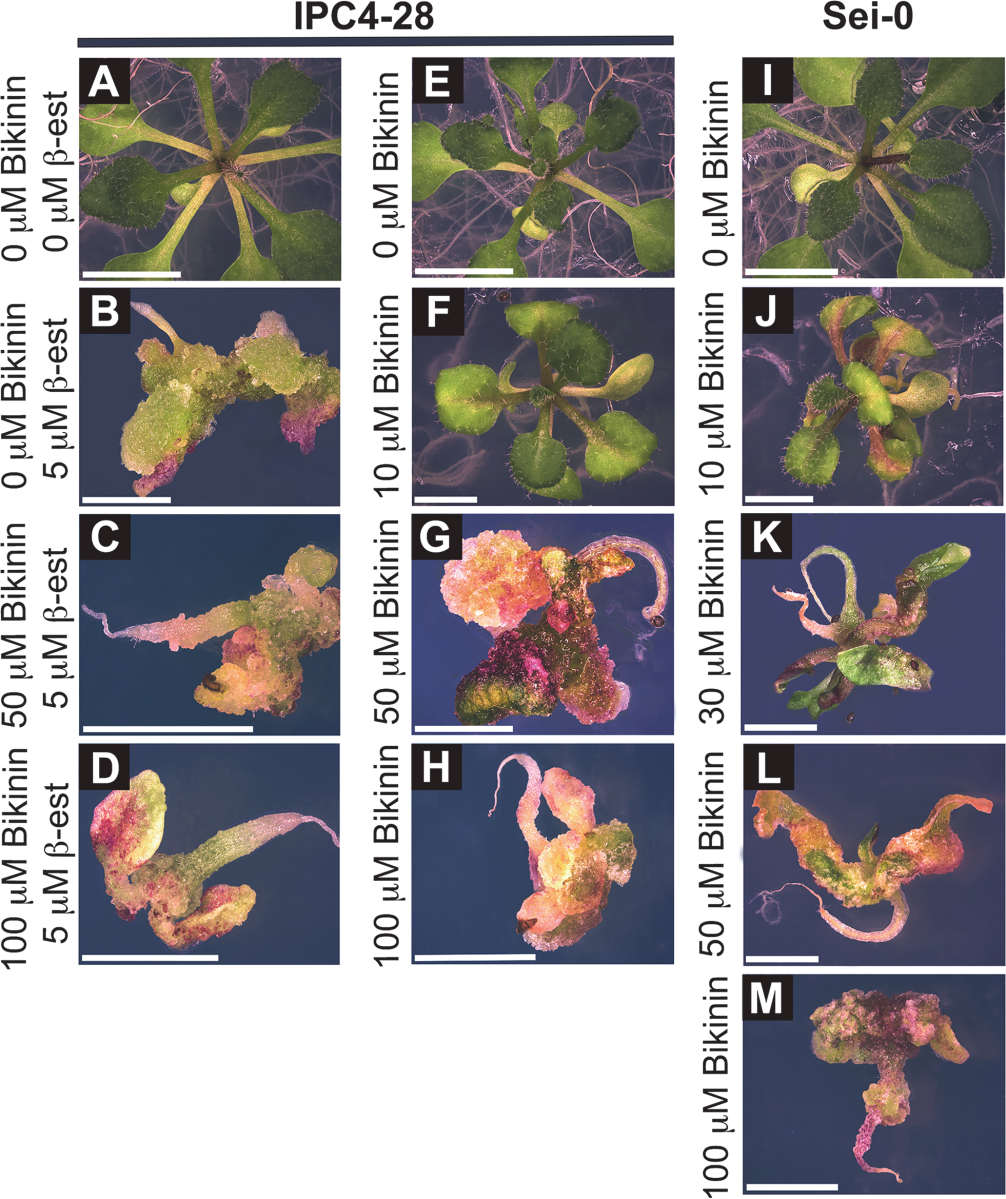
Bikinin induces hyperplasia. IPC4-28 seedlings germinated on solid induction media in the presence of increasing concentrations of bikinin (0 to 100 μM) at 20 days post germination (A-D). IPC4-28 seedlings (E-H) and Sei-0 seedlings (I-M) germinated on solid media amended with increasing concentrations of bikinin (0 to 100 μM) at 20 days post-germination. Scale bars = 5 mm (A, E, I) and 3 mm (B-D, F-H, and J-M).

Transgenic expression of C4 from an inducible promoter in *Arabidopsis* seedlings results in severe hyperplasia when seedlings are grown on induction media [9]. The lack of hyperplasia in a triple knockout mutant for sg2AtSKs, which displays a constitutive BR response [23,24], suggests that C4/sg2AtSK interactions are not solely responsible for the hyperplastic phenotype. To verify this, transgenic triple sg2AtSK mutant lines, Tmut132 and 152, were generated that express C4 under the regulatory control of the ß-estradiol inducible promoter. Tmut132 and Tmut152 seedlings grown in the absence of ß-estradiol in soil showed a phenotype that was indistinguishable from non-transgenic triple mutant seedlings (Fig 2A and 2C). Tmut132 and Tmut152 seedlings germinated on induction-media accumulated C4 protein (2J) and showed a hyperplastic phenotype similar to that of induced IPC4-28 seedlings (compare Fig 2E, 2F, 2H and 2I to Fig 3B) [9]. IPC4-28 is a transgenic *Arabidopsis* line that expresses C4 under the regulatory control of the ß-estradiol-inducible promoter [9]. Therefore, the C4 interaction with sg2AtSKs is not solely responsible for the C4 phenotype, suggesting that other C4/AtSK interactions may play a role.

The phosphorylation status of the BES transcription factor is a hallmark of BR signaling. Brassinolide (BL; the most active endogenous BR) inactivates AtSKs involved in BR regulation and induces nuclear accumulation of the active hypophosphorylated forms of BES1 and BZR1 [24,44,45]. The sg2AtSK triple mutant has significantly more hypophosphorylated BES1 relative to wild-type plants, but it retained the ability to accumulate much higher levels of phosphorylated BES1 than hypophosphorylated BES1, suggesting that a large pool of phosphorylated BES1 is under control of other AtSK family members [23,24]. The expression of BR-signaling target genes is altered in the presence of C4, in a manner similar to that following BL application [9], thus the expression of C4 should also alter the phosphorylation status of BES1. The phosphorylation status of BES1 was assayed in IPC4-28 seedlings induced with increasing concentrations of ß-estradiol, which were previously shown to positively correlate with increased levels of C4 protein [9]. Expression of C4 results in a shift from phosphorylated to hypophosphorylated BES1 with a positive correlation between the steady state levels of C4 protein and hypophosphorylated BES1 (Fig 2K). In contrast to the sg2AtSK triple mutant [23,24], the C4-expressing seedlings accumulate much higher levels of hypophosphorylated BES1 relative to phosphorylated BES1, suggesting that C4 is inhibiting sg2AtSKs and non-sg2AtSKs involved in BR signaling.

### Bikinin phenocopies C4-induced hyperplasia

Bikinin, an ATP competitor, which is an activator of BR signaling [33], selectively inhibits the kinase activity of 7 AtSK family members, including sg1AtSKs, sg2AtSKs and AtSK32 [25,33]; all of which were found to interact with C4 (Fig 1A). We hypothesized that if the C4 phenotype results from interactions with additional non-sg2AtSKs, then exposure to bikinin during seedling germination and growth might phenocopy the C4 phenotype and have little or no effect on the phenotype of C4-expressing seedlings. To determine if bikinin alters the phenotype induced by C4, we compared the phenotype induced when expressing C4 in the presence of increasing concentrations of bikinin. The hyperplasia observed in C4-expressing seedlings did not change with increasing concentrations of bikinin (compare Fig 3B to 3C and 3D). In the absence of ß-estradiol, 10 μM bikinin induced slight stunting and leaf rolling in IPC4-28 (Fig 3F) and Sei-0 seedlings (Fig 3J) reminiscent of a response to BL. However, higher concentrations of bikinin (≥30 μM) induced a hyperplasia in non-induced IPC4-28 and Sei-0 seedlings similar to the C4 phenotype in induced IPC4-28 seedlings (compare Fig 3G, 3H and 3K–3M to 3B). The bikinin-induced hyperplasia in Sei-0 seedlings was concentration-dependent (Fig 3J and 3L; S3 Table). These data suggest that bikinin inhibition of a set of AtSKs induces hyperplasia and that C4 may interact with and inactivate the same set of AtSKs, resulting in hyperplasia.

### Phosphoacceptor residue at position 49 is important for C4 function

**Fig 4:**
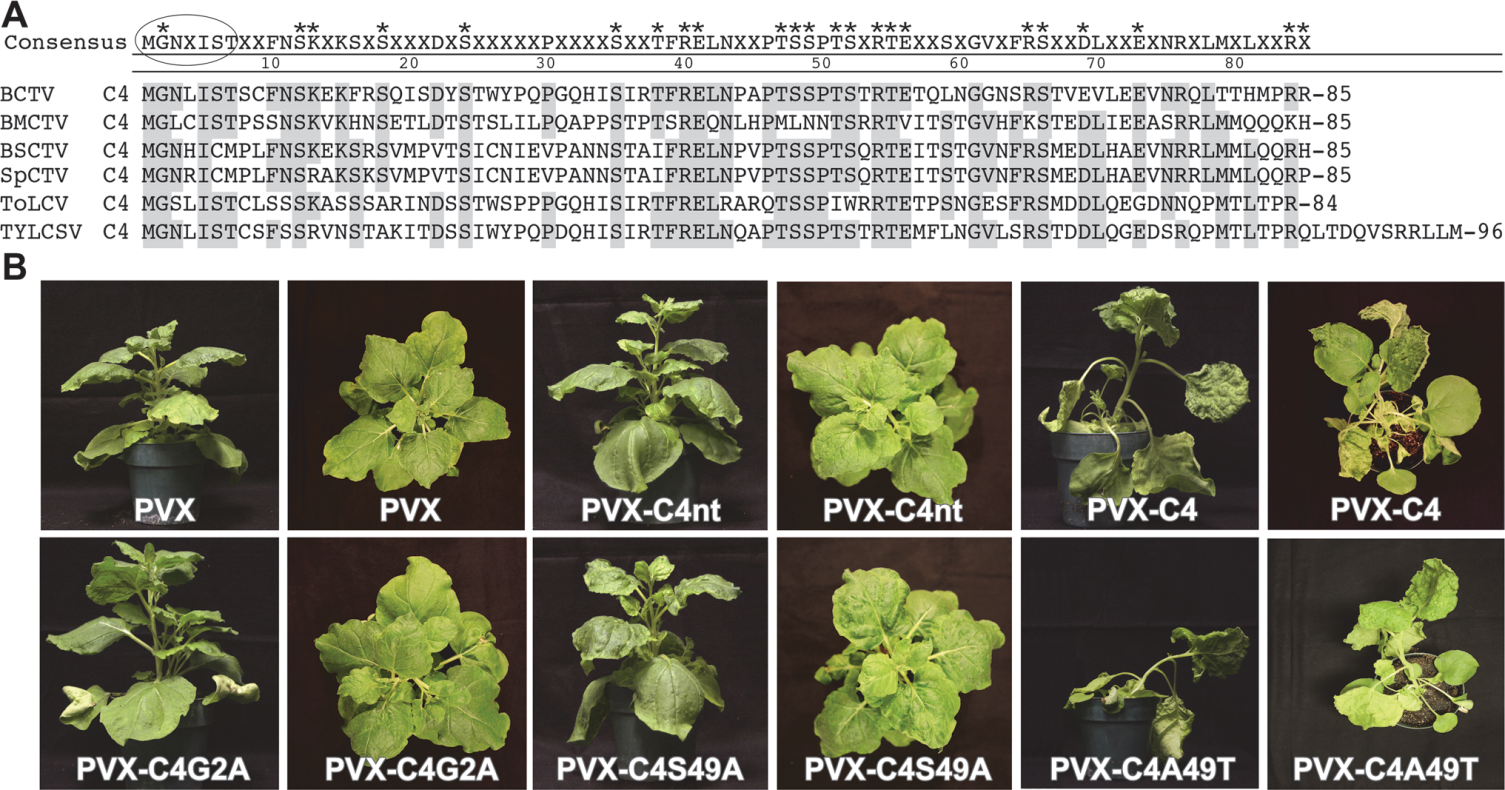
Phenotypic screening of C4 mutants using *N*. *benthamiana*. A. Alignment of C4 proteins from BCTV, BMCTV, BSCTV, SpCTV, ToLCV and TYLCSV. Asterisks indicate conserved amino acids that were mutated. N-myristoylation motif is indicated by an oval. B. Wild type *C4* or C4 mutants (*C4G2A*, *C4S49A*, *C4A49T*) or a non-translatable version of *C4* (*C4nt*) were cloned into a Potato virus X expression vector (pPVX). The pPVX derived *in vitro* transcripts were inoculated onto *N*. *benthamiana* plants. Phenotype induced by transcripts from pPVX-C4 represents the positive control. Phenotypes of *N*. *benthamiana* plants inoculated with transcripts from pPVX and pPVX-C4nt represent negative controls. Plants shown at 14 days post inoculation.

To identify amino acids critical for C4 function, alanine-scanning mutagenesis [46] was performed on amino acid residues that are conserved between the C4 proteins of four closely related curtoviruses, BCTV, *Beet mild curly top virus* (BMCTV-[US:Wor]), *Beet severe curly top virus* BSCTV-[US:Cfh], *Spinach curly top virus* (SpCTV-[US:Sp3:36]), and two monopartite begomoviruses, *Tomato yellow leaf curl virus* (TYLCV-IL[ES:Alm:Pep:99]) and ToLCV-To (Fig 4A). The C4 mutants were screened for the phenotype they induced on *N*. *benthamiana* plants following inoculation with genomic transcripts from a potato virus X based vector (pPVX) [34] carrying *C4* or the *C4*-mutants. A non-translatable *C4* (*C4nt*) served as a negative control [9]. All charged amino acid mutants except C4K13A retained a C4-like phenotype characterized by a contorted stem, rolling petioles, and epinastic, rough, bumpy and chlorotic leaves (Table 1). pPVX-C4K13A transcripts induced a mild C4-like phenotype (Table 1 and S3 Fig), but the mutant was not studied further.

AtSK21 phosphorylated C4 *in vitro* at undefined serine and threonine residues [17]. To identify putative phosphorylated residues in C4 required for function, conserved serine (Ser) and threonine (Thr) residues were mutated to alanine (Ala) (Table 1; Fig 4A). Conserved residues Ser12, -18, -49, -52 and Thr47 were predicted to have a high probability of being phosphorylated by NetPhos 2.0 analysis [47]. However, only substitutions at Ser18 and Ser49 affected the C4 phenotype (Table 1); C4S18A induced a mild C4-like phenotype while C4S49A induced a PVX-like phenotype (S3 Fig and Fig 4B, respectively). All other mutants retained a C4-like phenotype (Table 1). If phosphoacceptor amino acids at positions 18 and 49 are required for function, we posited that Thr, which can substitute for Ser as a substrate for phosphorylation, might restore the C4 phenotype. Thr was substituted for Ala in both C4S18A and C4S49A. Thr49 (C4A49T) rescued the C4 phenotype in *N*. *benthamiana* plants, whereas the Thr18 (C4A18T) phenotype remained the same (Fig 4B and Table 1, respectively).

**Fig 5:**
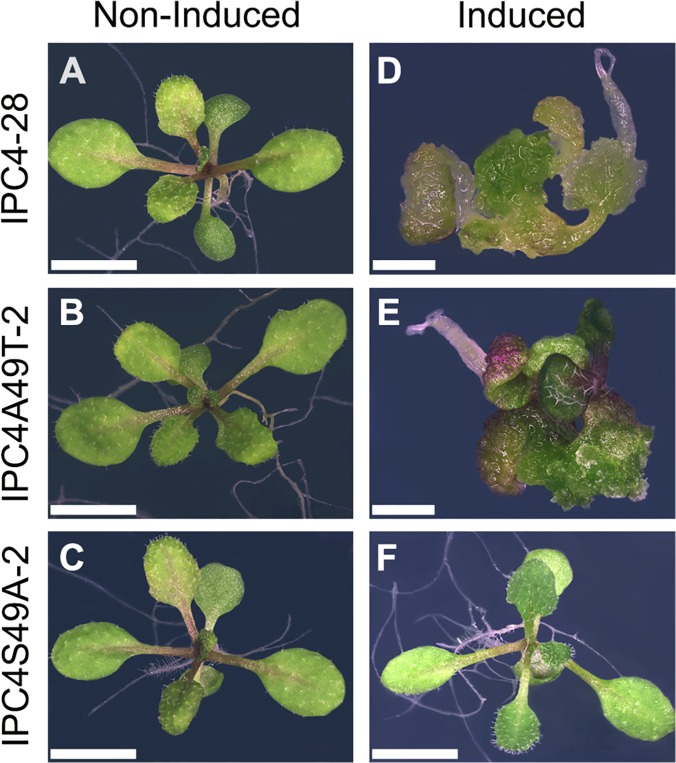
Serine 49 in the C4 protein is required for phenotype induction in transgenic seedlings. Transgenic *Arabidopsis* seedlings expressing C4 (IPC4-28) or C4 Ser49 mutants (IPC4S49A-2 and IPC4A49T-2) under the control of an inducible promoter were germinated on solid media (A, B, C) or solid induction media (D, E, F). IPC4-28 (A, D), IPC4A49T-2 (B, E), IPC4S49A-2 (C, F). Scale bar = 4 mm (A, B, C, F) and 2 mm (D, E). Seedlings shown at 15 days post induction.

To further confirm the necessity of a phosphoacceptor residue at amino acid residue 49 for C4 function, we generated transgenic *Arabidopsis* plants with *C4S49A* or *C4A49T* under regulatory control of the ß-estradiol inducible promoter. Homozygous, single-copy insertion transgenic lines expressing C4S49A (IPC4S49A-2) or C4A49T (IPC4A49T-2) at levels similar to wild-type C4 in transgenic line IPC4-28 (see below) were chosen for further study. IPC4S49A-2 and IPC4A49T-2 phenotypes, with and without ß-estradiol induction, were compared to transgenic line IPC4-28. Seedlings of the three lines developed normally and were indistinguishable from wild-type *Arabidopsis* when germinated in the absence of the inducer (Fig 5A and 5C). Seedlings of line IPC4S49A-2 germinated on induction media showed a phenotype that was indistinguishable from non-induced seedlings (compare Fig 5F with 5A and 5C), indicating a loss of C4 function. In contrast, IPC4A49T-2 seedlings showed a phenotype similar to that of IPC4-28 seedlings when germinated in the presence of the inducer (compare Fig 5D and 5E). Therefore, the C4S49A mutation abolishes the C4 phenotype in *Arabidopsis* and the C4A49T mutation restores the wild-type phenotype. Taken together, the results indicate that a phosphoacceptor residue at position 49 is required for function.

### *In vivo* mapping of C4 phosphorylation sites

To identify phosphorylated residues in C4 and C4A49T, the strepII tag was fused to the C-termini of the C4 and C4A49T proteins to give C4SII and C4A49TSII. The proteins, under the regulatory control of the ß-estradiol inducible promoter (see IPC4SII in S4A Fig), were expressed, extracted from transgenic plant tissue, and affinity purified (S4B Fig). Affinity purified proteins were subjected to mass spectrometry (MS). MS/MS and computational analysis confirmed that the proteins extracted from SDS-PAGE gel bands were C4SII and C4A49TSII. The analysis revealed that Ser21 of C4SII and C4A49TSII, Ser49 of C4SII, and Thr49 of C4A49TSII were phosphorylated (Table 2) with phosphoRS probability scores of 99–100% in three independent samples of each protein. Manual inspection of the MS/MS spectra confirmed the position of the phosphorylated residues in C4SII and C4A49TSII (S5 Fig).

**Table 2:** C4 phosphopeptides.

Sample	Amino Acid	M (Da)	Phosphopeptide sequence
C4/C4A49T	Ser21	2442.1005	17-RSQIpSDYSTWYPQPGQHISIRT-38
C4	Ser49	1536.6697	40-RELNPAPTSpSPTSTRT-55
C4A49T	Thr49	1550.6977	40-RELNPAPTSpTPTSTRT-55

*In vivo* phosphorylated C4SII or C4A49TSII proteins were expressed in transgenic plants, affinity purified from plant extracts, and analyzed by MS. The positions of phosphorylated amino acids, the mass of the peptides, and the phosphorylated peptide sequences are indicated.

### A phosphoacceptor residue at amino acid position 49 of C4 and an active kinase domain on AtSKs are necessary for C4-AtSK interactions

**Fig 6:**
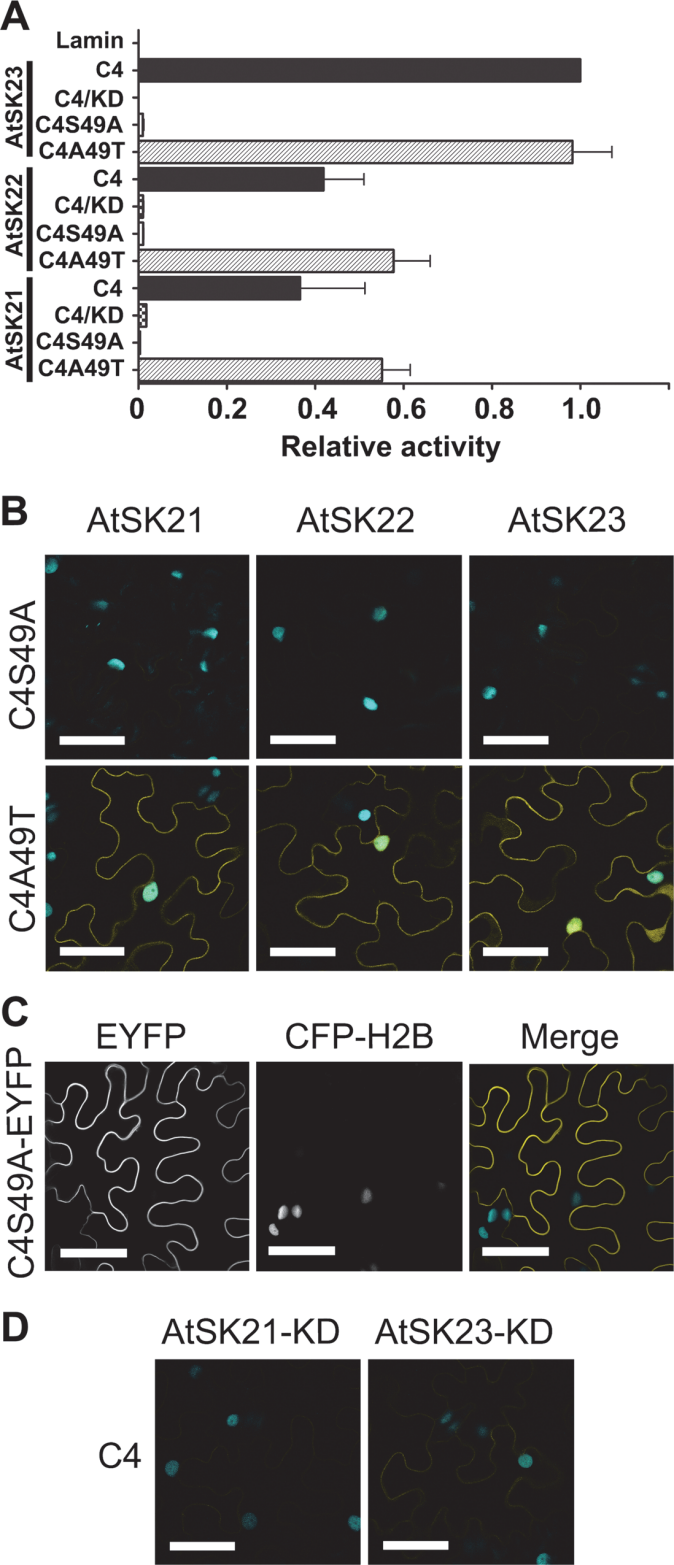
Phosphoacceptor residue in C4 and active kinase function in AtSKs are required for C4/AtSK interactions. A. Comparative strength of yeast two-hybrid interactions between C4 protein and wild-type or kinase dead (KD) mutants of the sg2AtSK family members, and between C4 mutants (C4S49A and C4A49T) and wild-type sg2AtSKs measured using ß-galactosidase activity. ß-galactosidase activity was quantified using triplicate samples from three individual yeast colonies for each interaction studied and is expressed relative to the C4/AtSK23 positive control interaction. The interaction between C4 and Lamin C is included as a negative control. B. Confocal micrographs of the interaction assay between the C4 Ser49 mutants (CS49A-cEYFP and C4A49T-cEYFP) and sg2AtSK-nEYFPs following co-agroinfiltration into transgenic *N*. *benthamiana* CFP-H2B marker plants. C. Confocal micrograph of C4S49A fused to EYFP following agroinfiltration into transgenic *N*. *benthamiana* CFP-H2B marker plants. D. Confocal micrographs of the interaction assay between C4-cEYFP and kinase dead (AtSK21-KD or AtSK23-KD) mutants following co-agroinfiltration into transgenic *N*. *benthamiana* CFP-H2B marker plants. The nuclear reference CFP fluorescence appears in blue and the interaction assay EYFP signal appears in yellow in the merged image (B). Scale bar = 50 μm.

To determine if a phosphoacceptor residue at position 49 is required for C4/AtSK interactions, we analyzed the interaction of C4, C4S49A and C4A49T with sg2AtSKs in yeast-two hybrid assays and *in planta* using BiFC assays. The importance of the phosphorylation of Ser21, a non-conserved residue, was not pursued in the present study. In yeast two-hybrid assays, C4A49T interacted with sg2AtSKs at levels similar to the wild-type C4 protein, whereas C4S49A failed to interact with sg2AtSKs (Fig 6A). The C4S49A mutant also failed to interact with sg2AtSKs *in planta* (Fig 6B). Co-expresssion of the C4S49A-cEYFP and AtSK21-nEYFP proteins was confirmed by immunoblotting (S2 Fig). Notably, C4S49A-EYFP localized to the plasma membrane, but was not detected in the nucleus in CFP-H2B plants (Fig 6C). The reversion mutant, C4A49T, interacted with sg2AtSKs *in planta* and localized to the plasma membrane and nuclei in a manner that was indistinguishable from C4/sg2AtSK interactions (compare Fig 1B and Fig 6B).

To assess whether a functional AtSK kinase activity was important for C4/AtSK interactions, we analyzed the interaction of C4 with kinase-dead (KD) mutants of the sg2AtSKs. The catalytically inactive AtSK21K69R mutant (referred to hereafter as AtSK21-KD) was unable to phosphorylate BZR1 [44,48]. We generated AtSK21-KD and mutated the homologous KD residues in AtSK22 and AtSK23 (AtSK22K99R and AtSK23K101R, referred to hereafter as AtSK22-KD and AtSK23-KD, respectively). No interaction was detected between C4 and sg2AtSK-KD mutants in yeast two-hybrid assays (Fig 6A). Similarly, no, or a very weak, interaction was detected between C4-cEYFP and AtSK21-KD-nEYFP or AtSK23-KD-nEYFP, respectively, in BiFC assays *in planta* (Fig 6D). The expression of C4-cEYFP and AtSK21-KD-nEYFP or AtSK23-KD-nEYFP in inoculated CFP-H2B plants was confirmed by immunoblot analysis (S2 Fig). Taken together, these data indicate that the presence of a phosphoacceptor site at amino acid residue 49 on C4 and an active kinase domain on AtSKs are necessary for C4 and AtSKs interactions.

### Phosphorylation of amino acid residue 49 is necessary for C4 modification of BR signaling

**Fig 7:**
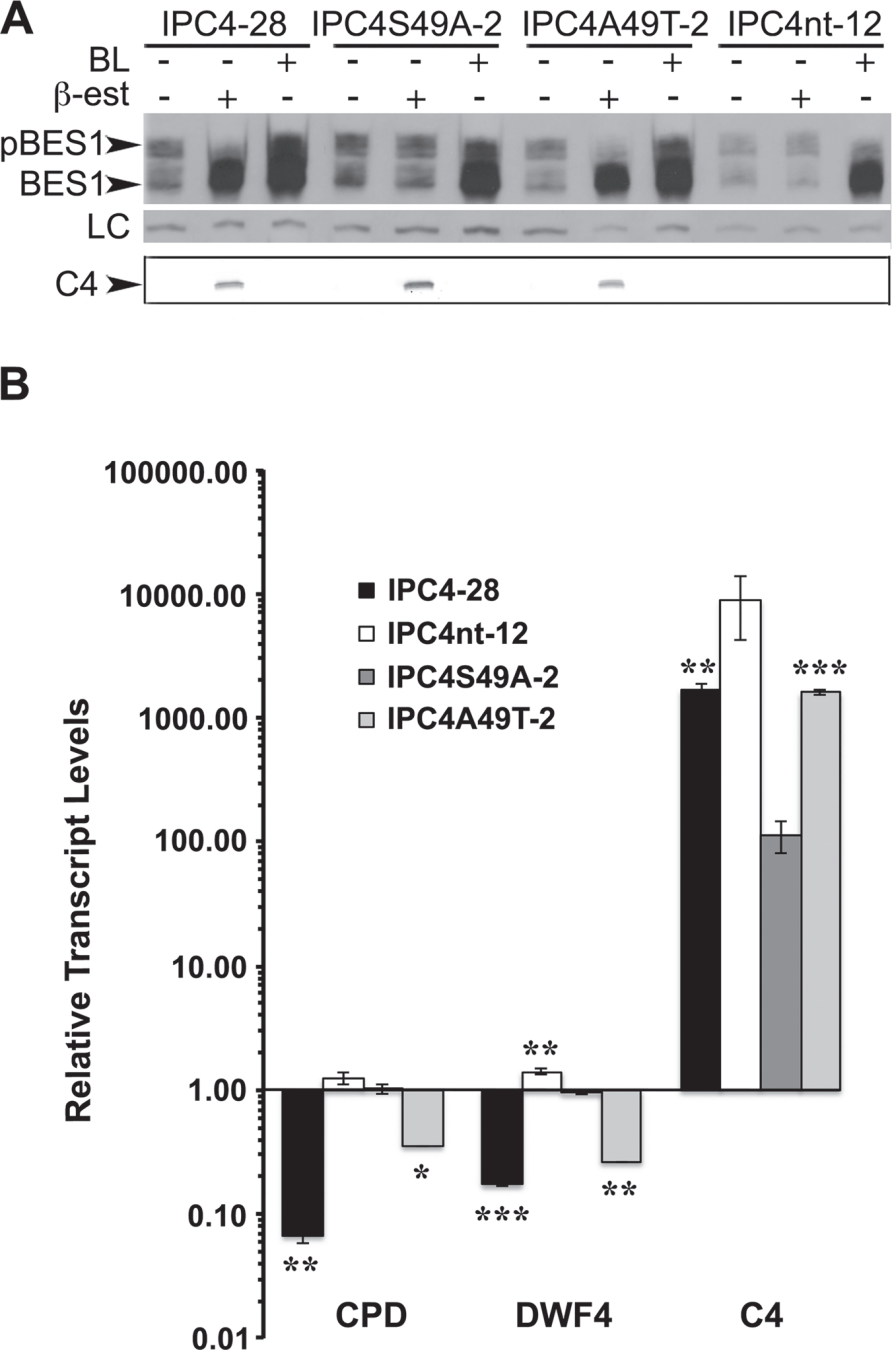
C4S49A mutation abolishes effect of C4 on BR signaling pathway. IPC4-28, IPC4S49A-2, IPC4A49T-2, and IPC4nt-12 seedlings were germinated and grown for 7 days in liquid media prior to induction and collected at 24 hpi. Total proteins and total RNA were extracted. A. Immunoblot analysis showing phosphorylation status of BES1 from non-induced, ß-estradiol or BL induced IPC4-28, IPC4S49A-2, IPC4A49T-2, or IPC4nt-12 seedlings (upper panel). Non-specific protein used as a loading control (LC) is shown in the middle panel. Induced expression of the C4 and C4 mutant proteins are shown in the lower panel. B. Quantitative real time PCR analysis. Transcript steady-state levels relative to non-induced samples for each gene are indicated. Transcript abundance was normalized to *ACT2*. Data represents means ±SD for two independent biological replicates. Asterisks indicate significant differences in the expression of IPC4, IPC4A49T, or IPC4nt compared to IPC4S49A (*ρ<0.05; **ρ<0.01; ***ρ<0.001) for each gene. BL, brassinolide; ß-est., ß-estradiol; phosphorylated BES1, pBES1, hypophosphorylated BES1, BES1.

To determine the biological significance of the loss of C4 function, we assayed the effect of C4 expression on BR signaling. Expression of C4 resulted in a shift between phosphorylated and hypophosphorylated BES1 levels, and a positive correlation between the steady state levels of C4 protein and hypophosphorylated BES1 (Fig 2K). We hypothesized that since C4S49A fails to interact with sg2AtSKs the level of phosphorylated BES1 and hypophosphorylated BES1 in seedlings expressing C4S49A should be similar to that of seedlings expressing C4nt. In contrast, seedlings expressing wild-type C4 and the C4A49T mutant should have decreased levels of phosphorylated BES1 and increased levels of hypophosphorylated BES1. In the absence of ß-estradiol and BL, all seedling lines showed similar amounts of phosphorylated and hypophosphorylated BES1 (Fig 7A). However, in the presence of ß-estradiol, BES1 was predominantly detected in the hypophosphorylated form in IPC4-28 and IPC4A49T-2 seedlings (Fig 7A). The shift from phosphorylated to hypophosphorylated BES1 was not detected in induced IPC4S49A-2 or IPC4nt-12 seedlings. As expected, in the presence of BL alone, all seedlings showed an increase in the levels of hypophosphorylated BES1 and a concomitant decrease in phosphorylated BES1 levels (Fig 7A). Interestingly, seedlings expressing the C4 and C4A49T proteins showed a greater decrease in phosphorylated BES than seedlings grown in the presence of BL. The data indicates that functional C4 inhibits phosphorylation of BES1 by possibly acting on AtSKs, and suggests that C4 is as effective at blocking BES1 phosphorylation as BL.

To further confirm the role of C4 in altering BR-signaling, we used qRT-PCR analysis to determine the expression levels of BR-signaling pathway target genes in seedlings expressing C4S49A. We previously showed that BZR1 target genes, *CPD* and *DWF4*, are repressed in the presence of the C4 protein [9] suggesting that C4 interferes with AtSK-directed phosphorylation and inactivation of BZR1. Therefore, expression of the non-functional C4S49A mutant protein should have no effect on *CPD* and *DFW4* transcript steady state levels. In contrast, the reversion mutant, C4A49T, should mimic C4. *CPD* and *DWF4* transcript levels were compared in induced IPC4-28, IPC4S49A-2, IPC4A49T-2 and IPC4nt-12 seedlings relative to non-induced seedlings by qRT-PCR at 24 h post-induction (Fig 7B). Steady state levels of *CPD* and *DWF4* transcripts were lower (>40.0- and >9.0-fold, respectively) in the presence of wild-type C4, while no significant changes in the expression levels of *CPD* and *DWF4* (<1.5-fold) were detected in the presence of C4S49A (Fig 7B). As expected, levels of *CPD* and *DWF4* transcripts were lower (>7.0- and >8.0-fold, respectively) in the presence of the revertant, C4A49T. These results corroborate the conclusion from Fig 7A and suggest that phosphorylation of residue 49 is required for C4 disruption of AtSK-dependent BR-signaling.

### Functional C4/AtSK interactions occur at the plasma membrane

**Fig 8:**
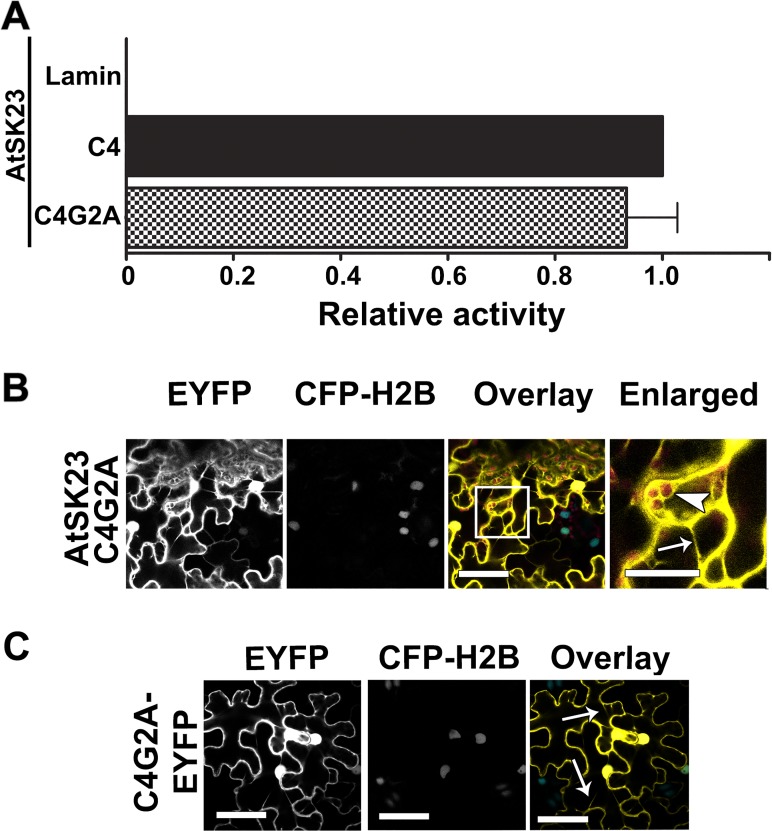
C4G2A interaction with sg2AtSKs *in planta* is localized to the cytosol and nucleus. A. Comparative strength of the interactions between C4 protein and C4G2A interactions with AtSK23. ß-galactosidase activity was quantified using triplicate samples from three individual yeast colonies and is expressed relative to the C4/AtSK23 positive control interaction. The interaction between C4 and Lamin C is included as a negative control. B. Confocal micrographs of the interaction between mutant C4G2A-cEYFP with AtSK23-nEYFP following co-agroinfiltration into CFP-H2B marker plants. From left to right, the first column shows the interaction assay (EYFP), the second column shows the nuclear signal from the reference marker (CFP-H2B), the third shows the merger between the two preceding panels (Overlay) and the fourth an enlargement of the boxed region in the Overlay. The co-localized EYFP signal appears in yellow, nuclear reference CFP fluorescence appears in blue, and the chloroplast autofluorescence is shown in red. C. Confocal micrograph of C4G2A fused to EYFP following agroinfiltration into transgenic *N*. *benthamiana* CFP-H2B marker plants. Arrowhead, chloroplasts; arrows, cytoplasmic strands. Scale bar = 50 μm.

A mutation in the myristoylation motif at the N-terminus of the C4 protein resulted in mislocalization of the mutant protein from the plasma membrane to the cytosol and nucleus and in loss of function [17]. To determine if the C4/AtSK interactions require C4 localization to the plasma membrane, we looked at the ability of a myristoylation mutant (C4G2A), which substitutes Ala2 for the myristoylated Gly2, to interact with AtSK23. The C4G2A mutant showed a phenotype that was indistinguishable from the phenotype induced by the negative control, C4nt, when inoculated into *N*. *benthamiana* (Fig 4B). The C4G2A protein interacted with AtSK23 similar to wild-type C4 in both yeast two-hybrid (Fig 8A) and BiFC assays (compare Fig 8B with Fig 1B). However, the fluorescent signal resulting from the C4G2A-cEYFP/AtSK23-nEYFP interaction was not localized to the plasma membrane, but rather was dispersed throughout the cytosol and nucleus, as confirmed by overlaying the autofluorescence (AF) of chloroplasts and fluorescence from CFP-H2B onto the EYFP signal from the C4G2A-cEYFP/AtSK23-nEYFP interaction. Unlike the C4-cEYFP/sg2AtSK-nEYFP interactions where the EYFP signal and chloroplast AF were distinct (Fig 1B), in the C4G2A-cEYFP/AtSK23-nEYFP interaction there was clearly an overlap between the EYFP and chloroplast AF signals (Fig 8B; Overlay and Enlarged images). The interaction between C4G2A-cEYFP and AtSK23-nEYFP was characterized by fluorescence in the cytosol surrounding chloroplasts, cytoplasmic strands, cytoplasmic granules, and in nuclei. Localization of the control fluorescent protein, C4G2A-EYFP, was indistinguishable from the C4G2A-cEYFP/AtSK23-nEYFP interaction (compare Fig 8B and 8C). The data indicate that the formation of C4/AtSK complexes do not require association with the plasma membrane, but that functional C4/AtSK interactions do require plasma membrane localization.

## Discussion

BCTV induces hyperplasia of phloem tissue and enations on the abaxial surface of veins in infected sugarbeet and *N*. *benthamiana* [8,49], while inducing extensive hyperplasia in infected *Arabidopsis* [10,50,51]. In contrast, BCTV containing C4 loss-of-function mutants failed to induce cell division, but remained infectious, illustrating the role of C4 in virus-induced hyperplasia, but not virus propagation [8,52]. More recently, expression of the C4 protein of BCTV in transgenic *Arabidopsis* was found to induce a severe development phenotype characterized by the loss of meristematic function, prolific cell division, and the loss of cell-type differentiation [9]. However, little is known about how C4 influences plant development and modulates the cell cycle. Here we show that the C4-induced phenotype is regulated by phosphorylation of Ser49 on plasma membrane localized C4. Our genetic and biochemical studies provide strong evidence that C4 interacts with seven members of the AtSK gene family. The interaction of C4 with sg2AtSKs and AtSK32, which regulate BR signal transduction (23,24), is consistent with the C4 protein altering the phosphorylation status of BES1 and in modulating the expression levels of BR-signaling target genes (SAUR-15, CPD and DWF4) (Fig 7) [9]. Bikinin, which inhibits the 7 AtSKs that C4 interacts with, phenocopies C4-induced hyperplasia, suggesting that the C4 protein and bikinin may interfere with the function of the same group, or sub-group, of AtSKs to induce hyperplasia.

Mass spectrometry analysis indicated that Ser21 and 49 were phosphorylated in C4 (Ser21 and Thr49 in C4A49T). While the importance of a phosphoacceptor residue at non-conserved residue 21 was not further pursued in this report, phosphorylation of a phosphoacceptor residue at position 49 was found to be required for C4-induced symptoms. Studies on recombinant BCTV C4 and TGMV AC4 proteins previously identified Ser49 as one of four amino acids in the central region of the protein that play a role in the C4 phenotype, although the significance of each individual amino acid was not determined [17]. In C4, Ser-49 precedes Pro-50 and represents a proline-directed Ser/Thr kinase phosphorylation motif (Ser/Thr-Pro). Ser/Thr-Pro motifs are major regulatory phosphorylation sites that function in a diverse array of cellular processes including cell cycle regulation, signal transduction and development [53]. GSK-3s, and by analogy the AtSK homologues, are members of a large family of proline-directed Ser/Thr kinases [53]. Because of its unique structure, proline can exist in either a *cis* or *trans* conformation, while most peptide bonds adopt the more energetically favored *trans* isomer. Phosphorylation of Ser/Thr-Pro motifs limit the rate of *cis/trans* isomerization and likely plays an important role in regulating protein structure and function [53]. Future analysis will better define the role of the phosphorylation site at residue 49 in C4 function and provide details of the mechanism involved in activating C4.

Mutants of C4 that disrupt C4/AtSK interactions (C4S49A) or localization of C4 to the plasma membrane (C4G2A) fail to induce hyperplasia or alter seedling development. The biological importance of the C4/AtSK interactions at the plasma membrane was supported by the following findings. First, the lack of a detectable interaction between plasma membrane localized C4S49A and AtSK23 *in planta* correlates with the loss of phenotype following induction of C4S49A in transgenic plants, the loss of the C4-induced phenotype when C4S49A was expressed from a chimeric PVX-vector following inoculation onto *N*. *benthamiana* plants, the inability of C4S49A to affect the phosphorylation status of phosphorylated BES1 and the inability to modify BZR1 target gene (*CPD* and *DWF4*) transcript steady state levels. Second, C4 and C4A49T interactions with sg2AtSKs localize to the plasma membrane and the nucleus, while the mutant C4G2A interacts strongly with AtSK23 in the cytosol and nucleus *in planta*, but does not localize to the plasma membrane and fails to induce a phenotype. Therefore, only C4/AtSK interactions at the plasma membrane are functionally important.

Our results show that bikinin, which specifically inhibits sg1AtSKs, sg2AtSKs and AtSK32 [33], induces hyperplasia in *Arabidopsis*. In contrast, BL, which negatively regulates the BR response by inhibiting sg2AtSK and AtSK32 activities [23,24,25], has not been reported to induce hyperplasia. While, the simultaneous inhibition of multiple AtSKs by bikinin may result in hyperplasia, it is plausible that bikinin inhibition of sg1AtSK members alone result in the phenotype. Alternatively, it is possible that the differing phenotypes induced by bikinin and BL result from the distinct mechanisms used by bikinin and BL to disrupt the balance between BR signal transduction and BR production. Bikinin binds directly to and inhibits AtSK kinase activity, while BL functions extracellularly at the plasma membrane to bind BRI1 and regulate the BR signal transduction pathway. Differential effects on the regulation of BR signal transduction by bikinin and BR is plausible given the plasticity of the BR-signaling pathway as illustrated in the observation that BR synthesis and BRI1-mediated signaling differentially control cell division and expansion [54].

The observed C4/AtSK interactions suggest that the C4 protein, similar to bikinin, binds directly to the same seven AtSKs, which could explain the phenocopying induced by C4 and bikinin. Bikinin induces hyperplasia in a concentration dependent manner with significant hyperplasia being observed at ≥30 μM bikinin. At bikinin concentrations of ≥50 μM, bikinin-induced hyperplasia was indistinguishable from C4-induced hyperplasia. Previously, bikinin (30 μM) was shown to induce significant developmental changes by 3 days post treatment [33] and to inhibit meristem development in 8-day-old seedlings [55], a change also seen in the presence of the C4 protein [9]. A recent study of bikinin-like inhibitors showed that bikinin was inactivated by conjugation with glutamic acid or malic acid [56], suggesting that concentrations of bikinin at ≥30 μM might be needed to see long term effects. This is supported by an IC_50_ of 23.3 μM for bikinin in a hypocotyl elongation assay (56).

There is considerable complexity in the role of AtSKs in a variety of biological processes [57]. However, much of the research to date has focused on the biochemical and genetic role of sg2AtSKs, and especially AtSK21, in regulating brassinosteroid signaling and in understanding the increasingly complex brassinosteroid network: a network that includes crosstalk between BR and other hormone signaling pathways, as well as the light signaling pathways [58,59,60]. In addition, the archetypical AtSK21 continues to be implicated in new roles such as regulating auxin signaling during lateral root development [61], stomatal development [62] and xylem cell differentiation [63]. In contrast, very little, if any, information is available about the roles of sg1, sg3, or sg4AtSKs, although expression data indicates that the function of AtSK family members has diversified with the development of distinct functions [57,64]. The extensive involvement of AtSK21 in regulating an array of cellular functions suggests that other AtSKs likely have extensive and diverse regulatory roles as well, which raises the question of what and how many AtSK roles the C4 protein usurps that might result in the loss of cell cycle control? The results suggest that the C4 protein interacts with 7 of the 10 AtSK family members, including the 4 that negatively regulate BR signaling, although it is possible that all seven of the AtSKs that C4 interacts with will be found to be involved in regulating BR signaling. It is tempting to speculate that one or more of the seven AtSKs that interact with C4 may either not be involved in BR signaling or, as mentioned above, evolved to have additional regulatory roles aside from BR signaling that impacts cell cycle control. Indeed, the lack of hyperplasia in transgenic sg2AtSK triple mutants unless C4 is expressed suggests that a C4 interaction with one or more members of sg1AtSKs and/or AtSK32 may play a primary role in the induction of hyperplasia.

The C4 protein provides an invaluable tool for expanding our understanding of the host factors and regulatory pathways involved in cell cycle regulation and to provide insights into the relationship between the plant cell cycle and development. In this study, we provide evidence that C4 function is activated by phosphorylation of Ser49 by members of the AtSK family and suggest that the protein regulates multiple AtSK activities that may subsequently disrupt mitotic control. Our results are consistent with a model in which C4 functions by sequestering multiple members of the AtSK family to the plasma membrane or by competing with AtSK target proteins resulting in inhibition of an array of AtSK functions. The possible disruption of AtSK function(s) by C4, which might equate to a similar response by bikinin, results in hyperplasia. Alternatively, an intriguing possibility is that C4 has an as yet undefined function after being activated by phosphorylation, which may modulate one or more signal transduction pathways at the plasma membrane, that result in hyperplasia. Future studies aimed at defining the specific C4/AtSKs interactions that result in hyperplasia and the role of phosphorylation in C4 function will reveal additional insights into the mechanism by which the C4 protein regulates cell division.

## Supporting Information

S1 Fig Phenotypic screening of C4-nEYFP and C4-cEYFP in *N*. *benthamiana* and localization of C4-EYFP and sg2AtSK-mCherry. A. Phenotypes induced following inoculation of transcripts from pPVX-C4-nEYFP (N-terminal portion of EYFP fused to the C-terminus of C4) or pPVX-C4-cEYFP (C-terminal portion of EYFP fused to the C-terminus of C4) onto *N*. *benthamiana*. For comparison, phenotypes of transgenic plants expressing the C4nt negative control and the C4 positive control. Third systemic leaf collected from plants expressing C4nt, C4, C4-cEYFP, or C4-nEYFP shown in the bottom panel. B. Confocal micrographs of C4 fused to EYFP agroinfiltrated into *N*. *benthamiana* CFP-H2B marker plants. C. Confocal micrographs of AtSK22, AtSK23, and AtSK41 fused to mCherry agro-infiltrated into *N*. *benthamiana* CFP-H2B plants. AtSK22-, AtSK23- and AtSK41-mCherry fusion proteins localize to the cytoplasm, evidenced by cytoplasmic strands (arrow) and cytoplasmic granules (arrowhead), and plasma membrane. The mCherry signal also co-localized to the nucleus with the CFP reference nuclear signal (CFP and overlay). Arrowhead, cytoplasmic granule; arrow, cytoplasmic strands. Scale bar = 50 μM.
(TIF)

S2 Fig Expression of C4-cEYFP, C4S49A-cEYFP and AtSK-nEYFPs following co-agroinfiltration into *N*. *benthamiana* during BiFC assays. Non-specific protein used as a loading control (LC) is shown below each immunoblot. Mw: AtSK21 = 43.01 kDa, AtSK23 = 46.53 kDa, AtSK41 = 47.68 kDa.
(TIF)

S3 Fig Phenotypic screening of C4 mutants in *N*. *benthamiana*. Phenotypes induced following inoculation of transcripts from pPVX-C4 mutants onto *N*. *benthamiana* plants. Transcripts from pPVX-C4 were used as a positive control. Transcripts from pPVX-C4nt and pPVX were used as negative controls. Plants shown at 14 days post inoculation. pPVX, potato virus X expression vector.
(TIF)

S4 Fig Expression and affinity purification of IPC4SII and IPC4A49TSII for MS. A. IPC4SII transgenic seedlings grown on solid media (Non-Induced) or solid induction media (Induced) at 12 days post-induction. Scale bars = 4 mm (Non-induced) and 2 mm (Induced). B. Immunoblot of extracts from induced IPC4-28, IPC4SII-6 and IPC4A49TSII-1 seedlings prior to affinity chromatography (Pre-affinity column) or following affinity chromatography (Post-affinity column). Non-specific protein used as a loading control (LC) is shown in the lower panel of Pre-affinity Column samples. Non-specific proteins were not present in Post-affinity Column samples. Mw: C4 = 9.69 kDa, C4SII = 10.89 kDa, C4A49TSII = 10.90 kDa.
(TIF)

S5 Fig Tandem mass spectrometric analyses of phosphopeptides observed in this study. A-C are the Collisional Induced Dissociation MS/MS spectra of SQIpS^21^DYSTWYPQPGQHISIR, ELNPAPTSpS^49^PTSTR, and ELNPAPTSpT^49^PTSTR, respectively. Their precursor ions are shown at the top of each spectrum. The key fragment ions are emphasized at the sequence diagrams for ambiguous phosphorylation site assignments.
(TIF)

S1 Table Primer pairs used for cloning. (DOCX)

S2 Table Primer pairs used for mutagenesis. (DOCX)

S3 Table Bikinin induced hyperplasia. (DOCX)
