# Supplementary figures and images for: The Roles of Phosphorylation and SHAGGY-Like Protein Kinases in Geminivirus C4 Protein Induced Hyperplasia
Source: PLoS One. 2015 Mar 27;10(3):e0122356. doi: 10.1371/journal.pone.0122356 (PMC4376871; doi:10.1371/journal.pone.0122356)

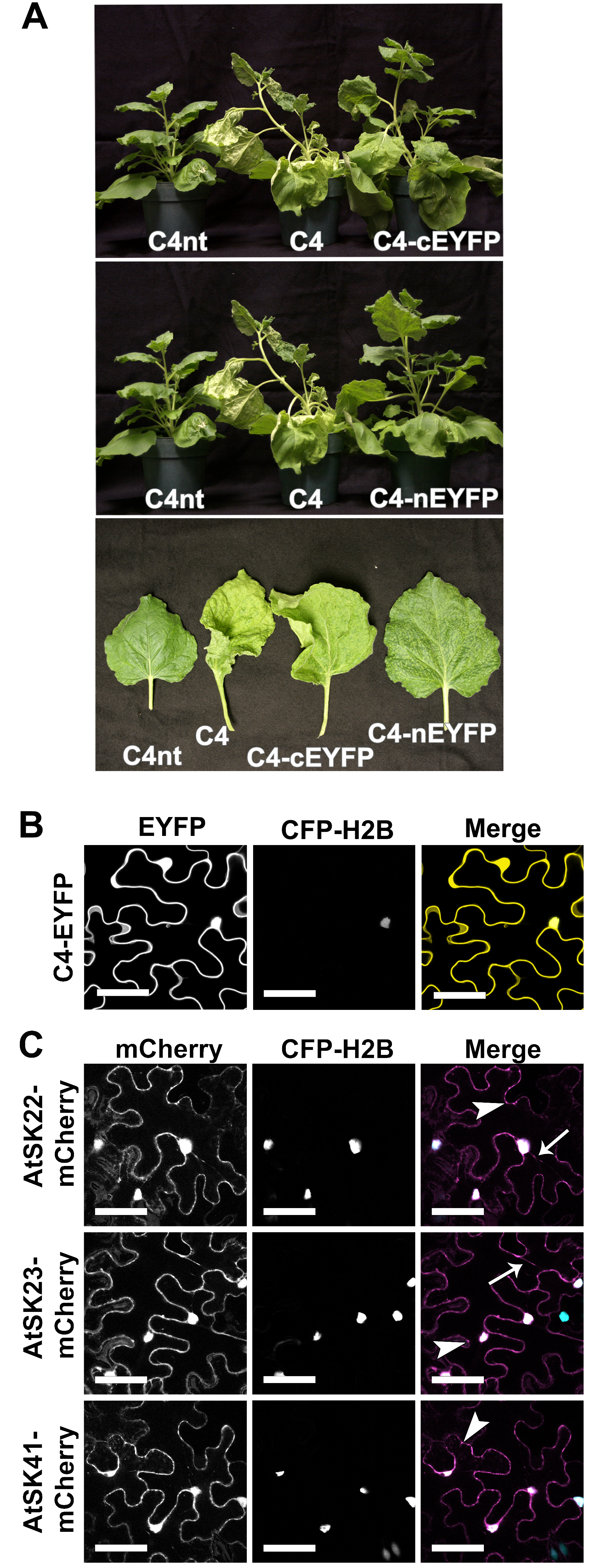

Supplement: S1 Fig — A. Phenotypes induced following inoculation of transcripts from pPVX-C4-nEYFP (N-terminal portion of EYFP fused to the C-terminus of C4) or pPVX-C4-cEYFP (C-terminal portion of EYFP fused to the C-terminus of C4) onto N. benthamiana. For comparison, phenotypes of transgenic plants expressing the C4nt negative control and the C4 positive control. Third systemic leaf collected from plants expressing C4nt, C4, C4-cEYFP, or C4-nEYFP shown in the bottom panel. B. Confocal micrographs of C4 fused to EYFP agroinfiltrated into N. benthamiana CFP-H2B marker plants. C. Confocal micrographs of AtSK22, AtSK23, and AtSK41 fused to mCherry agro-infiltrated into N. benthamiana CFP-H2B plants. AtSK22-, AtSK23- and AtSK41-mCherry fusion proteins localize to the cytoplasm, evidenced by cytoplasmic strands (arrow) and cytoplasmic granules (arrowhead), and plasma membrane. The mCherry signal also co-localized to the nucleus with the CFP reference nuclear signal (CFP and overlay). Arrowhead, cytoplasmic granule; arrow, cytoplasmic strands. Scale bar = 50 μM. (TIF) [file pone.0122356.s001.tif]

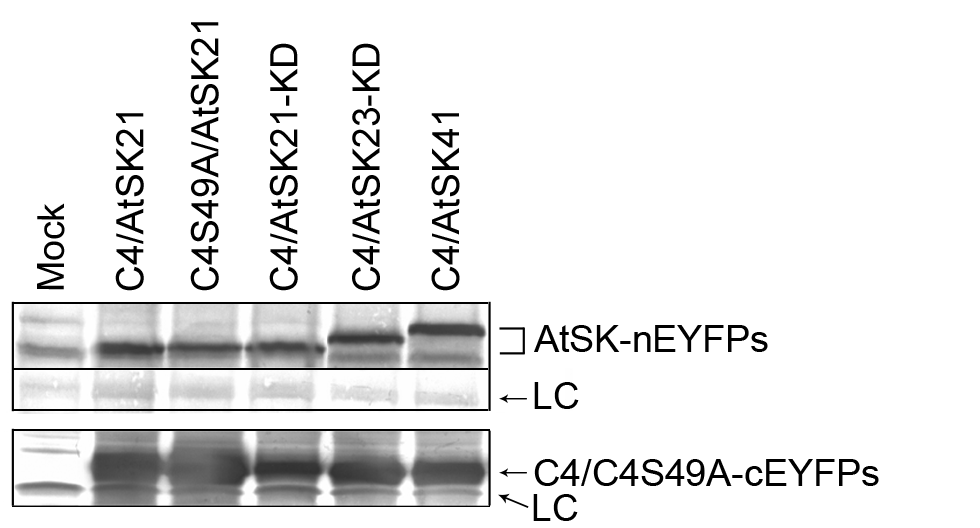

Supplement: S2 Fig — Non-specific protein used as a loading control (LC) is shown below each immunoblot. Mw: AtSK21 = 43.01 kDa, AtSK23 = 46.53 kDa, AtSK41 = 47.68 kDa. (TIF) [file pone.0122356.s002.tif]

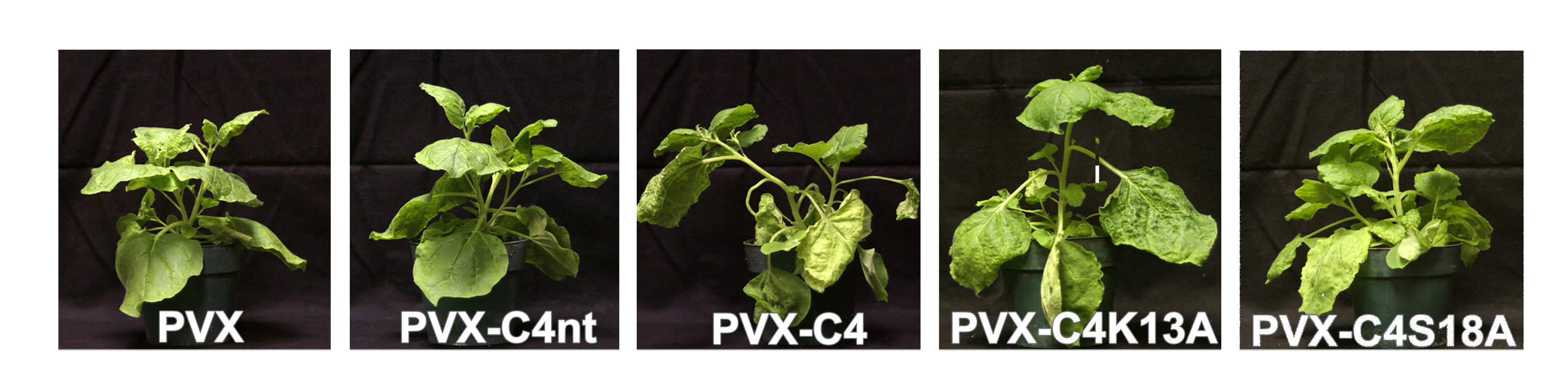

Supplement: S3 Fig — Phenotypes induced following inoculation of transcripts from pPVX-C4 mutants onto N. benthamiana plants. Transcripts from pPVX-C4 were used as a positive control. Transcripts from pPVX-C4nt and pPVX were used as negative controls. Plants shown at 14 days post inoculation. pPVX, potato virus X expression vector. (TIF) [file pone.0122356.s003.tif]

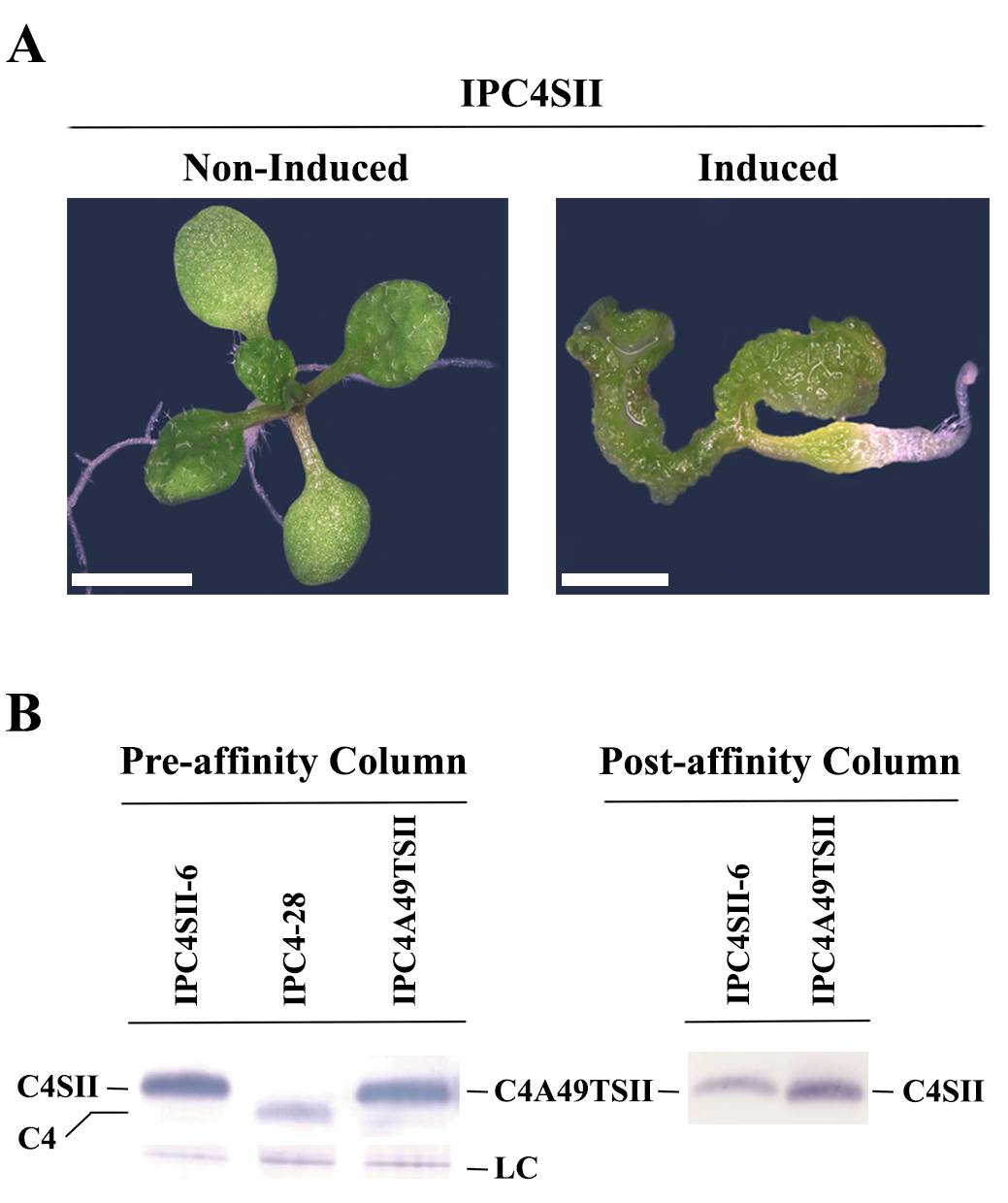

Supplement: S4 Fig — A. IPC4SII transgenic seedlings grown on solid media (Non-Induced) or solid induction media (Induced) at 12 days post-induction. Scale bars = 4 mm (Non-induced) and 2 mm (Induced). B. Immunoblot of extracts from induced IPC4-28, IPC4SII-6 and IPC4A49TSII-1 seedlings prior to affinity chromatography (Pre-affinity column) or following affinity chromatography (Post-affinity column). Non-specific protein used as a loading control (LC) is shown in the lower panel of Pre-affinity Column samples. Non-specific proteins were not present in Post-affinity Column samples. Mw: C4 = 9.69 kDa, C4SII = 10.89 kDa, C4A49TSII = 10.90 kDa. (TIF) [file pone.0122356.s004.tif]

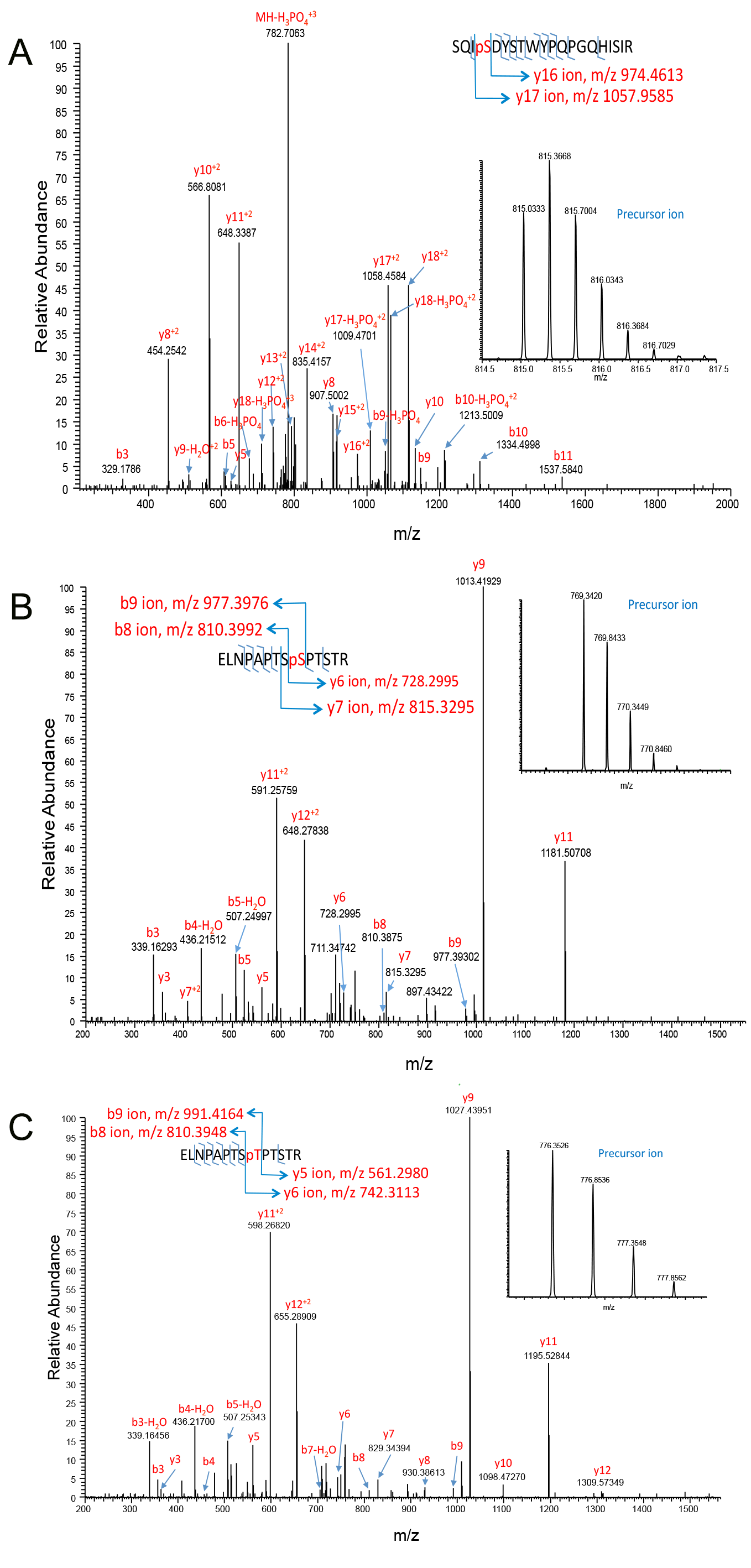

Supplement: S5 Fig — A-C are the Collisional Induced Dissociation MS/MS spectra of SQIpS21DYSTWYPQPGQHISIR, ELNPAPTSpS49PTSTR, and ELNPAPTSpT49PTSTR, respectively. Their precursor ions are shown at the top of each spectrum. The key fragment ions are emphasized at the sequence diagrams for ambiguous phosphorylation site assignments. (TIF) [file pone.0122356.s005.tif]
