# Supplementary material for: The Roles of Phosphorylation and SHAGGY-Like Protein Kinases in Geminivirus C4 Protein Induced Hyperplasia
Source: PLoS One. 2015 Mar 27;10(3):e0122356. doi: 10.1371/journal.pone.0122356 (PMC4376871; doi:10.1371/journal.pone.0122356)
Supplement: S1 Table — (DOCX) [file pone.0122356.s006.docx]

Table S1. Primer pairs used for cloning.

| **Primer name** | **Primer Sequence^1^** | **RE^2^** | **Target gene** |
| --- | --- | --- | --- |
| PVXC45 | CCGATCGATAACAATGGGCAACCTCATCTCCACGTCC | C | BCTV C4 |
| PVXC43 | CCGGATATCTTAACGCCTTGGCATATGAGTCG | E |  |
| C4NTE103 | CGGGATATCACGCCTTGGCATATGAGTCG | E | BCTV C4 |
| C4G2ApER5 | CGGCTCGAGAACAATGGCCAACCTCATCTCC | X | C4G2A |
| cEYFP EcoRev | CCGGATATCTCACTTGTACAGCTCGTCCATGC | E | C4-cEYFP |
| nEYFP EcoRev | CCGGATATCTCAGTCCTCGATGTTGTGGCGGA | E | C4-nEYFP |
| C4-EYFP 5F | CCGCCATGGGCAACCTCATCTCCACG | Nc | BCTV C4 |
| C4-EYFP 3R | CCGGGATCCCACGCCTTGGCATATGAGTCGT | B |  |
| BIN2-EYFP 5F | CCGCCATGGCTGATGATAAGGAGATG | Nc | AtSK21 (At4g18710)^3^ |
| BIN2-EYFP 3R | CCGGGATCCCAGTTCCAGATTGATTCAAGAA | B |  |
| SK22longEcoF | CCGGAATTCATGGCCTCATTACCATTGGGG | E | AtSK22  (At1g06390)^3^ |
| SK22BamHI3R | CCGGGATCCTTAACTGTTTTGTAATCCTGT | B |  |
| SK23-EYFP 5F | CCGAATTCATGACTTCGATACCATTGGGG | E | AtSK23 (At2g30980)^3^ |
| SK23BamHI3R | CCGGGATCCCTAGGGTCCAGCTTGAAATGG | B |  |
| SK41-EY 5F | CCGCTCGAGATGGCATCCTCTGGACTGGGA | E | AtSK41 (At1g09840)^3^ |
| SK41BamHI3R | CCGGGATCCTTACGAATGCAAAGCCATGAA | B |  |
| At3g24240 5F | CCGGAATTCATGTCTCTTCATTCCTTAATC | E | LRR-RLK (At3g24240)^3^ |
| At3g24240 3R | CCGGGATCCCCTCGATTGAAGAAGAAGAAGA | B |  |
| AtSK11 H3 5F | CCGAAGCTTATGGCGTCAGTGGGTATAGCT | H | AtSK11  (At5g26751)^3^ |
| AtSK11 B 3R | CCGGGATCCCCAAACCGAGCCAAGGACACT | B |  |
| AtSK12 H3 5F | CCGAAGCTTATGGCCTCGGTGGGCATAGA | H | AtSK12 (At3g05840)^3^ |
| AtSK12 B 3R | CCGGGATCCCCAAACTGAGCCACGGACATT | B |  |
| AtSK13 N 5F | CCGCCATGGCTTCTGTGGGAACATTA | Nc | AtSK13 (At5g14640)^3^ |
| AtSK13 B 3R | CCGGGATCCCGAGAGCGAGGAAGGAACATT | B |  |
| AtSK31 N 5F | CCGGAATTCATGAATGTGGTGCGGAGATTA | Nc | AtSK31 (At3g61140) ^3^ |
| AtSK31 B 3R | CCGGGATCCCTTTCCTTGCATGCTCAGGTA | B |  |
| AtSK32 N 5F | CCGAGATCTATGAACGTGATGCGTCGTCT | Nc | AtSK32 (At4g00720) ^3^ |
| AtSK32 ERI 3R | CCGGAATTCAGAGCTACTTCCCGTTCCCT | E |  |
| SK42 Nco5F | CCGCCATGGAATCTCATCTGGGAAATGGA | Nc | AtSK42 (At1g57870)^3^ |
| AtSK42 B 3R | CCGGGATCCCCGAATGCAAAGCCATGAAGA | B |  |
| BaitC45PR | CGCCATGGGCAACCTCATCTCCACG | Nc | BCTV C4 |
| BDC43PR | CGGGATCCTTAACGCCTTGGCATATGAGTCG | B |  |
| C4G2A5Nco | CGCCATGGCCAACCTCATCTCCACG | Nc | C4G2A |
| 72StpBamHI | CCGGGATCCTTAACGCCTTGGCATATGAGT | B | C4-71Stp |
| SK22BamHI3R | CCGGGATCCTTAACTGTTTTGTAATCCTGT | B | AtSK22 ^4^ |
| SK23BamHI3R | CCGGGATCCCTAGGGTCCAGCTTGAAATGG | B | AtSK23 ^4^ |
| SK41BamHI3R | CCGGGATCCTTACGAATGCAAAGCCATGAA | B | AtSK41 ^4^ |
| LRRBamHI 3R | CCGGGATCCTCACTCGATTGAAGAAGAAGA | B | LRR-RLK ^4^ |
| AtSK11 Nde 5F | CCGCATATGGCGTCAGTGGGTATAGCT | N | AtSK11 ^5^ |
| SK11 B STP | CCGGGATCCTCACAAACCGAGCCAAGGACA | B |  |
| AtSK12 Nde 5F | CCGCATATGGCCTCGGTGGGCATAGA | N | AtSK12 ^5^ |
| SK12 B STP | CCGGGATCCTCACAAACTGAGCCACGGACA | B |  |
| SK13 B STP | CCGGGATCCTTAGAGAGCGAGGAAGGAACA | B | AtSK13 ^4^ |
| SK31 B STP | CCGGGATCCTCATTTCCTTGCATGCTCAGG | B | AtSK31 ^4^ |
| AtSK32 ERI 5F | CCGGAATTCATGAACGTGATGCGTCGTCT | E | AtSK32 ^5^ |
| AtSK32 Sal 3R | CCGGTCGACCTAAGAGCTACTTCCCGTTCC | S |  |
| AtSK42 N 5F | CCGCCATGGCATCCTCTGGACTGGGA | Nc | AtSK42 ^5^ |
| SK42 B STP | CCGGGATCCTTACGAGTGTAATGCCATGAA | B |  |
| LOGANC43SII | CGGACTAGTTTATTTTTCGAACTGCGGGTGGCTCCAAGCGCTACGCCTTGGCATATGAGTCGTTAGCTG | S | C4; C4S49T |

^1^ Restriction site is underlined.

^2^ RE = restriction enzyme used for cloning. B, *Bam*HI; C, *Cla*I; E, *Eco*RI; H, *Hind*III; N, *Nde*I; Nc, *Nco*I; S, *Sal*I; *Spe*I; X, *Xho*I.

^3^ Primer pair for cloning gene into pSAT vectors for BiFC.

^4^ For cloning gene into pGBKT7 for yeast two-hybrid. This 3’ primer used in combination with 5’ primer for pSAT vector cloning.

^5^ Primer pair for cloning gene into pGBKT7 for yeast two-hybrid.
