# Supplementary material for: The Roles of Phosphorylation and SHAGGY-Like Protein Kinases in Geminivirus C4 Protein Induced Hyperplasia
Source: PLoS One. 2015 Mar 27;10(3):e0122356. doi: 10.1371/journal.pone.0122356 (PMC4376871; doi:10.1371/journal.pone.0122356)
Supplement: S2 Table — (DOCX) [file pone.0122356.s007.docx]

Table S2. Primer pairs used for mutagenesis.

| **Primer name** | **Primer sequence** | **Mutant^1^** |
| --- | --- | --- |
| C4G2AF | ATCGATAACAATGGCCAACCTCATCTCC | C4G2A |
| C4G2AR | GGAGATGAGGTTGGCCATTGTTATCGATACC |  |
| C4S12AF | TCCTGCTTCAACGCGAAGGAAAAGTTCAG | C4S12A |
| C4S12AR | GAACTTTTCCTTCGCGTTGAAGCAGGACG |  |
| C4K13AF | TGCTTCAACTCGGCGGAAAAGTTCAGATC | C4K13A |
| C4K13AR | TCTGAACTTTTCCGCCGAGTTGAAGCAGG |  |
| C4S18AF | GAAAAGTTCAGAGCACAAATATCAGATTATTCG | C4S18A |
| C4S18AR | ATCTGATATTTGTGCTCTGAACTTTTCCTTCGA |  |
| C4S18EF | [GAAAAGTTCAGAGAACAAATATCAGATTATTCGACC](C4%20project/C4%20mutagenesis/primer%20design/phosp%20primer%20order%20form#%091,1440,1488,0,,GAA%09AAG%09TTC%09AGA%09GAA%09CAA%09ATA%09TCA%09) | C4S18E |
| C4S18ER | [ATAATCTGATATTTGTTCTCTGAACTTTTCCTTCGAG](C4%20project/C4%20mutagenesis/primer%20design/phosp%20primer%20order%20form#%091,1501,1550,0,,ATA%09ATC%09TGA%09TAT%09TTG%09TTC%09TCT%09GAA%09) |  |
| C4E18TF | GAAAAGTTCAGAACACAAATATCAGATTATTCGACC | C4S18T |
| C4E18TR | ATAATCTGATATTTGTGTTCTGAACTTTTCCTTCGAG |  |
| C4S24AF | ATATCAGATTATGCGACCTGGTATCCC | C4S24A |
| C4S24AR | GGATACCAGGTCGCATAATCTGATATTTG |  |
| C4S35AF | CAGCACATTGCCATCCGAACATTCAG | C4S35A |
| C4S35AR | GAATGTTCGGATGGCAATGTGCTGACC |  |
| C4R40F | CCGAACATTCGCGGAGCTAAATCCAGCTCCG | C4R40A |
| C4R40R | GCTGGATTTAGCTCCGCGAATGTTCGGATG |  |
| C4E41F | CGAACATTCAGGGCGCTAAATCCAGCTCC | C4E41A |
| C4E41R | TGGATTTAGCGCCCTGAATGTTCGGATGG |  |
| C4T47AF | CCAGCTCCGGCGTCAAGTCCTACG | C4T47A |
| C4T47AR | CGTAGGACTTGACGCCGGAGCTGG |  |
| C4S48AF | GCTCCGACGGCAAGTCCTACGTCG | C4S48A |
| C4S48AR | CGTAGGACTTGCCGTCGGAGCTGG |  |
| C4S49AF | GCTCCGACGTCAGCTCCTACGTCGACAAGG | C4S49A |
| C4S49AR | TGTCGACGTAGGAGCTGACGTCGGAGCTGG |  |
| C4S49EF | [GCTCCGACGTCAGAACCTACGTCGACAAGG](C4%20project/C4%20mutagenesis/primer%20design/phosp%20primer%20order%20form#%091,689,729,0,,GCT%09CCG%09ACG%09TCA%09GAA%09CCT%09ACG%09TCG%20) | C4S49E |
| C4S49ER | [TGTCGACGTAGGTTCTGACGTCGGAGCTGG](C4%20project/C4%20mutagenesis/primer%20design/phosp%20primer%20order%20form#%091,742,781,0,,TGT%09CGA%09CGT%09AGG%09TTC%09TGA%09CGT%09CGG%09) |  |
| C4A49TF | TCCGACGTCAACTCCTACGTCGACAAG | C4S49T |
| C4A49TR | TGTCGACGTAGGAGTTGACGTCGGAGC |  |
| C4T51AF | ACGTCAAGTCCTGCGTCGACAAGGAC | C4T51A |
| C4T51AR | CTTGTCGACGCAGGACTTGACGTCGG |  |
| C4S52AF | TCAAGTCCTACGGCGACAAGGACGG | C4S52A |
| C4S52AR | GTCCTTGTCGCCGTAGGACTTGACG |  |
| C4R54AF | CCTACGTCGACAGCGACGGAGACAC | C4R54A |
| C4R54AR | GTGTCTCCGTCGCTGTCGACGTAGGAC |  |
| C4T55AF | GTCGACAAGGGCGGAGACACAATTG | C4T55A |
| C4T55AR | CAATTGTGTCTCCGCCCTTGTCGACG |  |
| C4T55EF | [ACGTCGACAAGGGAGGAGACACAATTGATTGGG](C4%20project/C4%20mutagenesis/primer%20design/phosp%20primer%20order%20form#%091,1224,1267,0,,ACG%09TCG%09ACA%09AGG%09GAG%09GAG%09ACA%09CAA%09) | C4T55E |
| C4T55ER | [CAATTGTGTCTCCTCCCTTGTCGACGTAGG](C4%20project/C4%20mutagenesis/primer%20design/phosp%20primer%20order%20form#%091,1280,1319,0,,CAA%09TTG%09TGT%09CTC%09CTC%09CCT%09TGT%09CGA%09) |  |
| C4E56AF | ACAAGGACGGCGACACAATTGAATGGGG | C4E56A |
| C4E56AR | CAATTGTGTCGCCGTCCTTGTCGACG |  |
| C4R65AF | GGGGGGAATTCCGCATCGACGGTAGAAG | C4R65A |
| C4R65AR | CTACCGTCGATGCGGAATTCCCCCCATTC |  |
| C4S66AF | GGGAATTCCAGAGCGACGGTAGAAGTGC | C4S66A |
| C4S66AR | CTTCTACCGTCGCTCTGGAATTCCCCCC |  |
| C4E69AF | TCGACGGTAGCAGTGCTAGAGGAGG | C4E69A |
| C4E69AR | CTCTAGCACTGCTACCGTCGATCTGGA |  |
| C4E73AF | GTGCTAGAGGCGGTCAACAGACAGC | C4E73A |
| C4E73AR | GTCTGTTGACCGCCTCTAGCACTTCTAC |  |
| C4R84AF | TCATATGCCAGCGCGTTAAGATATCGAATTCC | C4R84A |
| C4R84AR | TATCTTAACGCGCTGGCATATGAGTCGTTAGC |  |
| C4R85AF | TATGCCAAGGGCTTAAGATATCGAATTCCTGC | C4R85A |
| C4R85AR | TTCGATATCTTAAGCCCTTGGCATATGAGTCG |  |
| C4R40AE41AF | TCCATCCGAACATTCGCGGCGCTAAATCCAGCTCCG | C4R40AE41A |
| C4R40AE41AR | AGCTGGATTTAGCGCCGCGAATGTTCGGATGGAAATGTG |  |
| C4S52AR54AF | ACGTCAAGTCCTACGGCGACAGCGACGGAGACACAA | C4S52AR54A |
| C4S52AR54AR | GTGTCTCCGTCGCTGTCGCCGTAGGACTTGACGTCG |  |
| C4E72AE73AF | GTAGAAGTGCTAGCGGCGGTCAACAGACAGC | C4E72AE73A |
| C4E72AE73AR | GTCTGTTGACCGCCGCTAGCACTTCTACCG |  |
| SK21K69R F | GAAACCGTGGCGATACGGAAGGTTTTGCAAGAT | AtSK21K69R |
| SK21K69R R | ATCTTGCAAAACCTTCCGTATCGCCACGGTTTC |  |
| SK22K99R F | GAATCAGTAGCCATTCGAAAGGTCTTGCAAGAT | AtSK22K99R |
| SK22K99R R | ATCTTGCAAGACCTTTCGAATGGCTACTGATTC |  |
| SK23K101RF | GAATCAGTAGCCATTCGGAAGGTTTTGCAAGAT | AtSK23K101R |
| SK23K101RR | ATCTTGCAAAACCTTCCGAATGGCTACTGATTC |  |

1. Mutant designation: Gene name(Original amino acid)(residue position)(New amino

acid).
