# Supplementary material for: The Roles of Phosphorylation and SHAGGY-Like Protein Kinases in Geminivirus C4 Protein Induced Hyperplasia
Source: PLoS One. 2015 Mar 27;10(3):e0122356. doi: 10.1371/journal.pone.0122356 (PMC4376871; doi:10.1371/journal.pone.0122356)
Supplement: S3 Table — (DOCX) [file pone.0122356.s008.docx]

**Table S3.** Bikinin induced hyperplasia.^1^

| Genotype | DPI^2^ | 10 µM  Bikinin | 30 µM  Bikinin | 50 µM  Bikinin | 100 µM  Bikinin | 200 µM  Bikinin |
| --- | --- | --- | --- | --- | --- | --- |
| Sei-0 | 10 | 6% (146)^3^ | 35% (136) | 45% (271) | 80% (407) | 99% (338) |
| Sei-0 | 20 | 8% (131) | 40% (141) | 55% (226) | 96% (513) | 100% (172) |

^1^% of seedlings showing hyperplasia at indicated bikinin concentrations.

^2^Days post-induction

^3^ Numbers in parentheses represents the total number of seedlings observed.
